# Federal Housing Assistance and Stage at Cancer Diagnosis Among Older Adults in the US

**DOI:** 10.1001/jamanetworkopen.2025.36281

**Published:** 2025-10-08

**Authors:** Craig Evan Pollack, Amanda L. Blackford, Taylor K. Craig, Qinjin Fan, S. M. Qasim Hussaini, Katherine L. Chen, Daniel Polsky, Margaret Katana Ogongo, Joan L. Warren, Cary P. Gross, K. Robin Yabroff

**Affiliations:** 1 Department of Health Policy and Management, Johns Hopkins Bloomberg School of Public Health, Baltimore, Maryland; 2 Johns Hopkins School of Nursing, Baltimore, Maryland; 3 Department of Medicine, Johns Hopkins University School of Medicine, Baltimore, Maryland; 4 Sidney Kimmel Comprehensive Cancer Center, Johns Hopkins Medicine, Baltimore, Maryland; 5 Division of Quantitative Sciences, Department of Oncology, Johns Hopkins University School of Medicine, Baltimore, Maryland; 6 Biostatistics, Epidemiology and Data Management Core, Johns Hopkins University School of Medicine, Baltimore, Maryland; 7 Surveillance, Prevention, & Health Services Research (SPHeRe), American Cancer Society, Atlanta, Georgia; 8 O’Neal Comprehensive Cancer Center, Division of Hematology & Oncology, Department of Medicine, University of Alabama at Birmingham, Birmingham; 9 Division of General Internal Medicine & Health Services Research, Department of Medicine, David Geffen School of Medicine, University of California, Los Angeles, Los Angeles; 10 Carey Business School, Johns Hopkins University, Baltimore, Maryland; 11 Department of Internal Medicine, Yale School of Medicine, New Haven, Connecticut; 12 Yale Cancer Outcomes, Public Policy, and Effectiveness Research Center, New Haven, Connecticut

## Abstract

**Importance:**

A growing number of older adults live in unaffordable and unstable housing; however, whether programs designed to counter housing insecurity contribute to earlier-stage cancer diagnosis remains largely unknown.

**Objective:**

To examine the association between the receipt of federal housing assistance, which limits household spending on rent and utilities, and cancer stage at diagnosis among older adults in the US.

**Design, Setting, and Participants:**

This comparative cohort study used the Surveillance, Epidemiology, and End Results cancer registry program and Medicare database linked with data from the US Department of Housing and Urban Development (HUD). Participants were individuals aged 66 to 95 years who received new diagnoses of breast cancer, colorectal cancer, non–small cell lung cancer (NSCLC), or prostate cancer between 2007 and 2019. Data were acquired in 2023, and the data analysis was performed from June 2023 through March 2025.

**Exposure:**

Receipt of federal housing assistance.

**Main Outcomes and Measures:**

The primary outcome was stage at cancer diagnosis, defined as in situ (for breast cancer only), localized, regional, or distant. Odds ratios (ORs) for the association between having HUD assistance at diagnosis and cancer stage at diagnosis were estimated using multinomial (nonproportional odds) regression. Individuals with HUD assistance were propensity score matched to individuals without housing assistance.

**Results:**

A total of 52 532 individuals (mean [SD] age at diagnosis, 76.3 [6.8] years; 33 608 women [64.0%]) with housing assistance at diagnosis were included: 16 064 had breast cancer, 10 807 had colorectal cancer, 17 156 had NCSLC, and 8505 had prostate cancer; 38 183 (72.7%) were enrolled in Medicaid, and 38 539 (73.4%) had Part D low-income cost sharing. Compared with matched controls, fewer individuals with housing assistance received a diagnosis of distant breast cancer (1071 patients [6.7%] vs 3485 patients [7.2%]; adjusted OR [aOR], 0.85; 95% CI, 0.82- 0.90), distant colorectal cancer (2398 patients [22.2%] vs 7562 patients [23.3%]; aOR, 0.90; 95% CI, 0.83-0.98), and distant NSCLC (8810 patients [51.4%] vs 27 901 patients [54.2%]; aOR, 0.83; 95% CI, 0.79-0.86) compared with localized cancers. Housing assistance was not significantly associated with stage at diagnosis for individuals with prostate cancer. The association between housing assistance and stage at cancer diagnosis varied across the different types of housing assistance, including the Housing Choice voucher program, multifamily housing, and public housing.

**Conclusions and Relevance:**

The findings of this cohort study of older adults with cancer suggest that federal housing assistance was associated with earlier-stage diagnosis of breast cancer, colorectal cancer, and NSCLC, highlighting its potential role in mitigating the adverse associations of housing insecurity with cancer outcomes.

## Key Points

**Question:**

Is there an association between receipt of federal housing assistance and stage at cancer diagnosis among older adults in the US?

**Findings:**

In this cohort study of 52 532 individuals receiving housing assistance who were propensity score matched to individuals receiving no assistance, those who received housing assistance were significantly less likely to receive a diagnosis of distant-stage breast cancer (6.7% vs 7.2%), colorectal cancer (22.2% vs 23.3%), and non–small cell lung cancer (51.4% vs 54.2%).

**Meaning:**

These findings suggest that policies designed to improve housing security are associated with reduced odds of late-stage diagnosis and may be an important step toward reducing cancer disparities.

## Teaser

This cohort study investigates the associations between receipt of federal housing assistance and the diagnosis of cancer at different stages among older adults in the US.

## Introduction

Across the US, approximately 6% of patients with breast, 8% with prostate cancer, 22% with colorectal cancer, and 43% with lung cancer receive an initial diagnosis at a late stage,^1^ leading to more intensive treatments, increased morbidity and mortality,^1,2^ and higher health care costs than if detected earlier.^3^ Variation in late-stage diagnosis contributes to the longstanding disparities in cancer mortality by race and socioeconomic status (SES).^1,4^

Housing insecurity, a growing crisis in the US, may be an underappreciated determinant of late-stage cancer diagnosis and disparities. Housing insecurity encompasses suboptimal housing affordability, stability, quality, and safety and neighborhood conditions.^5^ Racial and ethnic inequities in housing insecurity mirror similar inequities in cancer outcomes and stem, in large part, from a long history of systemic racism in housing policies.^6,7^ Housing insecurity can disrupt health care access and continuity by straining household budgets, which causes frequent moves, and by fueling stress, which is linked to cancer pathogenesis and progression.^8,9,10,11^ These health care interruptions may delay cancer detection and diagnosis. Senior adults aged 65 years or older are particularly at risk, given the higher cancer incidence and increasing rates of housing insecurity in this population, with 11.7 million seniors spending more than one-half their incomes on housing.^12^

Efforts to increase housing security may facilitate earlier cancer detection. Federal housing assistance reaches approximately 1.8 million seniors, limiting rent and utilities to 30% of income.^13^ Most recipients of federal housing assistance are Black (44%) and fall into the extremely low-income category, with average annual household incomes of $18 000 or lower.^13^ There are multiple types of housing assistance, including Housing Choice Vouchers, which allow households to rent homes on the private market, as well as public housing and multifamily housing, in which subsidies are tied to specific housing units. By improving housing affordability and stability, ensuring units meet quality standards through inspections, and expanding access to diverse neighborhoods and services, housing assistance may help facilitate earlier cancer diagnosis.^14^ Research examining the impact of housing assistance on cancer outcomes is limited.^15^ With only 1 in 4 households that are eligible for housing assistance actually receiving it due to limited program funding,^16^ research into the potential positive impact on health outcomes of housing assistance is urgently needed to inform policy and resource allocation.

Leveraging a novel data linkage, we assessed whether older adults in the US who received federal housing assistance were less likely to receive a diagnosis of late-stage cancer compared with those who did not receive housing assistance. We examined breast cancer, colorectal cancer, non-small cell lung cancer (NSCLC), and prostate cancer, as these are the 4 most commonly diagnosed nonskin cancers, have high morbidity and mortality, and have existing screening tests for early detection.

## Methods

### Study Design

Drawing from a sample of Medicare beneficiaries with a cancer diagnosis, this study used a longitudinal cohort design with propensity score matching to compare stage at diagnosis between individuals who received federal housing assistance at the time of their diagnosis with those who did not. The study followed Strengthening the Reporting of Observational Studies in Epidemiology (STROBE) reporting guidelines and was determined to be non–human participants research by the Johns Hopkins Bloomberg School of Public Health institutional review board and no informed consent was needed because data were deidentified.

### Data Sources

The Surveillance, Epidemiology, and End Results (SEER) cancer registry-Medicare data were linked with administrative records from the US Department of Housing and Urban Development (HUD).^17^ SEER registries report cancer information for approximately 48% of the US population. Medicare is the federal health insurance program, administered by the Centers of Medicare & Medicaid Services, for individuals who are aged 65 years and older, as well as any younger individuals who have end-stage kidney disease or a disability. The linked SEER-Medicare data include persons who received a cancer diagnosis from 2007 through 2019, along with their Medicare enrollment and fee-for-service claims from 2006 through 2020. HUD administrative data from 2005 to 2021 contain nationwide information on persons receiving federal housing assistance, including housing assistance type, duration, and household characteristics. HUD assistance receipt dates were used to determine when individuals received housing assistance relative to their cancer diagnosis.

### Study Population

The study population included individuals with a first primary cancer diagnosis between ages 66 and 95 years of breast cancer (female patients only), colorectal cancer, NSCLC, or prostate cancer (male patients only) between 2007 and 2019. Exclusion criteria included male breast cancer, autopsy or death certificate diagnosis, unknown month of diagnosis, incomplete Medicare enrollment information, or missingness in covariates used for matching, and stage, the primary end point.

### Outcome and Exposures

We defined stage at diagnosis according to SEER summary stage as in situ (breast cancer only), localized, regional, or distant. Individuals were classified as having HUD assistance at diagnosis if they were continuously enrolled in an HUD program from at least 6 months before diagnosis to diagnosis month, in order to ensure sufficient exposure to housing assistance in ways that might influence diagnostic care. All others were classified as not having HUD assistance at diagnosis and served as potential controls for propensity score matching.

### Covariates

Covariates were age at diagnosis, sex (colorectal cancer and NSCLC only), marital status, year of diagnosis, SEER registry, and metropolitan residence. Insurance characteristics included Medicaid or Medicare Advantage enrollment in diagnosis month and original reason for Medicare entitlement. Race and ethnicity, included to reflect social and structural characteristics including experiences with housing assistance, were defined by SEER from extracted medical records. The Yost deprivation index for an individual’s neighborhood (census tract) of residence was used as an area-based measure of SES and classified into state-based quintiles.

### Statistical Analysis

Data were acquired in 2023, and the data analysis was performed from June 2023 through March 2025. Characteristics of individuals with and without housing assistance were compared using standardized mean differences for each cancer type. Using the MatchIt package in R statistical software version 4.4.1 (R Project for Statistical Computing)^18^, individuals with HUD assistance were matched to individuals without housing assistance using propensity scores with a nearest neighbor distance metric in a 3-to-1 ratio without replacement. All covariates listed above were included in the matching, with specification of an exact match on age at diagnosis. We did not include interaction or higher-order terms in the model, and no caliper was specified. We assessed match quality by examining standardized mean differences and density plots of the covariates between individuals with HUD assistance and controls before and after matching.

Odds ratios (ORs) for the association between having HUD assistance at diagnosis and cancer stage—in situ (breast only), localized (reference), regional, or distant—at diagnosis were estimated using multinomial (nonproportional odds) regression. All models included the unweighted covariates used in the matching to yield doubly robust results in case of misspecification in either the propensity score or multinomial models. We used the marginaleffects R package to estimate the average treatment effect on the treated and 95% CIs and the MNLpred package to compute estimated probabilities.^19,20^

In secondary analyses, we examined whether the association between housing assistance and stage varied according to the following prespecified subgroups: age at diagnosis (66-74 years and 75-95 years), sex (male, female; colorectal cancer and NSCLC models only), race or ethnicity (American Indian and Alaska Native, Asian American and Pacific Islander, Hispanic, non-Hispanic Black, and White), neighborhood SES (Yost index state quintile), and time period relative to NSCLC screening introduction (2007-2014 vs 2015-2019) and changing prostate cancer screening guidelines (2007-2011, 2012-2017, or 2018-2019). To ensure covariate balance, the sample was rematched in each stratum, using the same matching process as described above.^21^ For the analyses that examined racial and/or ethnic variation, we used 2-to-1 matching rather than 3-to-1 matching due to the small sample size among some racial and/or ethnic groups. For each cancer type, heterogeneity of the estimated treatment effect across subgroups was tested using interaction terms between the subgroup category and receipt of housing assistance, with overall *P* values from analysis of deviance tests reported. We also ran models separately according to type of housing assistance (Housing Choice voucher, public housing, and multifamily housing), where we rematched each subgroup to its own control group. Because the comparison group did not have housing assistance, we did not perform interaction analyses and qualitatively evaluated differences across groups.

We performed sensitivity analyses to ensure the robustness of our findings. First, we examined the association between housing assistance and stage using the American Joint Committee on Cancer stage, which is missing for the period of this study for all individuals in the Idaho, Massachusetts, New York, and Texas SEER registries and some individuals in the other registries. Second, the primary analysis was unable to account for comorbidity, which can impact an individual’s likelihood of receiving screening and later-stage cancer diagnosis. We therefore performed an analysis limiting our sample to individuals with 12 months of fee-for-service Medicare claims history prior to diagnosis, from which we were able to calculate Charlson Comorbidity Index scores for use in propensity score matching. We also explored alternative specifications of the propensity score model, including 2-to-1 and 4-to-1 matching ratios under the nearest neighbor distance metric framework, as well as exploring exact and coarsened exact matching approaches. Next, we calculated E-values for the primary analysis of the association between receipt of federal housing assistance on stage at cancer diagnosis, for results that were found to be statistically significant (*P* < .05) for a 2-sided test.^22^ Finally, we applied published data on cancer incidence and costs,^3,23^ together with our estimated differences in the estimated probability of stage with vs without housing assistance, to estimate the potential health care cost savings associated with extending housing assistance to those households most in need.^24^

*P* < .05 was considered statistically significant for the primary analyses. For secondary analyses (heterogeneity of the outcome across subgroups), significance was adjusted using a Bonferroni threshold based on the total number of interactions tests by each cancer type: breast cancer (*P* < .05/4 = .0125), colorectal cancer (*P* < .05/5 = .01), NSCLC (*P* < .05/6 = .008), and prostate cancer (*P* < .05/5 = .01).

## Results

The total cohort included 1 477 032 individuals (eFigure 1 in Supplement 1). The study sample analyzed included 52 532 patients (33 608 women [64.0%]) with HUD assistance at diagnosis: 16 064 women with breast cancer, 10 807 individuals with colorectal cancer, 17 156 individuals with NSCLC, and 8505 men with prostate cancer (Table 1). Their mean (SD) age at diagnosis was 76.3 (6.8) years; 8703 (16.6%) were Hispanic, 13 494 (25.7%) were non-Hispanic Black, 25 590 (48.7%) were non-Hispanic White, 38 183 (72.7%) were enrolled in Medicaid, and 38 539 (73.4%) had Part D low-income cost sharing. Compared with patients without HUD assistance, those with HUD assistance were more likely to be female (colorectal cancer and NSCLC), non-Hispanic Black, not married, and living in metropolitan areas and in neighborhoods with the lowest SES. With regard to insurance coverage, people with HUD assistance were more likely to have had disability as an original reason for Medicare entitlement and were more likely to have Medicaid. After propensity score matching for each cancer type, standardized mean differences in all covariates were less than 0.20, indicating good balance between those with and without HUD assistance within each cancer type (eTable 1 and eFigure 2 in Supplement 1).

**Table 1.** Patient Characteristics at Cancer Diagnosis

Characteristic	Patients, No. (%)^a^							
	Breast cancer		Colorectal cancer		NSCLC		Prostate cancer	
	Not HUD assisted (n = 400 451)	HUD assisted (n = 16 064)^b^	Not HUD assisted (n = 246 090)	HUD assisted (n = 10 807)^b^	Not HUD assisted (n = 357 998)	HUD assisted (n = 17 156)^b^	Not HUD assisted (n = 419 961)	HUD assisted (n = 8505)^b^
Age, mean (SD), y	74.9 (6.7)	76.0 (6.8)	77.1 (7.3)	78.0 (7.2)	76.2 (6.7)	76.4 (6.7)	73.4 (5.8)	74.5 (6.1)
Sex								
Male	NA	NA	119 486 (48.6)	3719 (34.4)	182 153 (50.9)	6700 (39.1)	419 961 (100.0)	8505 (100.0)
Female	400 451 (100.0)	16 064 (100.0)	126 604 (51.4)	7088 (65.6)	175 845 (49.1)	10 456 (60.9)	NA	NA
Race and ethnicity								
Hispanic (all races)	33 867 (8.5)	3030 (18.9)	24 780 (10.1)	1734 (16.0)	22 186 (6.2)	1984 (11.6)	39 482 (9.4)	1955 (23.0)
Non-Hispanic American Indian and Alaska Native	1234 (0.3)	48 (0.3)	878 (0.4)	33 (0.3)	1138 (0.3)	57 (0.3)	1187 (0.3)	21 (0.2)
Non-Hispanic Asian American and Pacific Islander	20 107 (5.0)	1039 (6.5)	13 703 (5.6)	1119 (10.4)	17 861 (5.0)	1705 (9.9)	18 172 (4.3)	723 (8.5)
Non-Hispanic Black	35 385 (8.8)	4122 (25.7)	22 163 (9.0)	2399 (22.2)	29 303 (8.2)	4105 (23.9)	48 662 (11.6)	2868 (33.7)
Non-Hispanic White	309 858 (77.4)	7825 (48.7)	184 566 (75.0)	5522 (51.1)	287 510 (80.3)	9305 (54.2)	312 458 (74.4)	2938 (34.5)
Married^c^	118 160 (29.5)	1296 (8.1)	77 495 (31.5)	1348 (12.5)	111 551 (31.2)	1953 (11.4)	181 224 (43.2)	1591 (18.7)
Metropolitan residence^d^								
Large metropolitan	320 430 (80.0)	14 007 (87.2)	189 998 (77.2)	9319 (86.2)	273 333 (76.4)	14 482 (84.4)	326 997 (77.9)	7541 (88.7)
Medium or small metropolitan	32 748 (8.2)	923 (5.7)	20 623 (8.4)	610 (5.6)	32 190 (9.0)	1146 (6.7)	37 597 (9.0)	463 (5.4)
Rural	47 273 (11.8)	1134 (7.1)	35 469 (14.4)	878 (8.1)	52 475 (14.7)	1528 (8.9)	55 367 (13.2)	501 (5.9)
Original reason for Medicare entitlement								
Age	374 361 (93.5)	12 680 (78.9)	225 634 (91.7)	9030 (83.6)	315 410 (88.1)	13 018 (75.9)	386 837 (92.1)	6604 (77.6)
Disability with or without ESKD	26 090 (6.4)	3384 (21.1)	20 456 (8.4)	1777 (16.4)	42 588 (12.0)	4138 (24.2)	33 124 (7.9)	1901 (22.3)
Medicaid enrolled	45 718 (11.4)	11 593 (72.2)	38 594 (15.7)	7899 (73.1)	56 486 (15.8)	12 540 (73.1)	34 892 (8.3)	6151 (72.3)
Part D low-income cost sharing^e^								
Fully subsidized	46 526 (11.6)	11 695 (73.0)	38 805 (15.8)	7965 (73.9)	57 247 (16.0)	12 673 (74.0)	35 670 (8.5)	6206 (73.4)
Eligible, not receiving	8655 (2.2)	869 (5.4)	6570 (2.7)	550 (5.1)	9732 (2.7)	870 (5.1)	6528 (1.6)	439 (5.2)
Not eligible	344 429 (86.2)	3451 (21.5)	200 128 (81.5)	2270 (21.0)	290 449 (81.3)	3592 (21.0)	376 473 (89.9)	1813 (21.4)
Enrolled in Medicare Advantage	133 701 (33.4)	5794 (36.1)	78 886 (32.1)	3269 (30.2)	113 202 (31.6)	5633 (32.8)	130 693 (31.1)	3118 (36.7)
SEER Registry								
California	96 064 (24.0)	3241 (20.2)	57 468 (23.4)	2434 (22.5)	75 629 (21.1)	3356 (19.6)	95 551 (22.8)	1721 (20.2)
Connecticut	12 366 (3.1)	668 (4.2)	7194 (2.9)	386 (3.6)	11 524 (3.2)	730 (4.3)	13 216 (3.1)	360 (4.2)
Georgia	28 592 (7.1)	800 (5.0)	16 614 (6.8)	508 (4.7)	27 313 (7.6)	878 (5.1)	34 411 (8.2)	505 (5.9)
Hawaii	4840 (1.2)	130 (0.8)	3035 (1.2)	94 (0.9)	3773 (1.1)	164 (1.0)	5111 (1.2)	71 (0.8)
Idaho	5011 (1.3)	88 (0.5)	2877 (1.2)	38 (0.4)	4329 (1.2)	93 (0.5)	6118 (1.5)	32 (0.4)
Iowa	11 431 (2.9)	343 (2.1)	8458 (3.4)	246 (2.3)	10 996 (3.1)	398 (2.3)	12 889 (3.1)	93 (1.1)
Kentucky	14 989 (3.7)	535 (3.3)	11 135 (4.5)	363 (3.4)	19 294 (5.4)	820 (4.8)	15 001 (3.6)	187 (2.2)
Louisiana	14 496 (3.6)	449 (2.8)	10 057 (4.1)	258 (2.4)	14 980 (4.2)	517 (3.0)	18 944 (4.5)	294 (3.5)
Massachusetts	23 145 (5.8)	1558 (9.7)	12 872 (5.2)	973 (9.0)	21 135 (5.9)	1763 (10.3)	20 604 (4.9)	773 (9.1)
Detroit	12 731 (3.2)	532 (3.3)	8026 (3.3)	375 (3.5)	13 202 (3.7)	682 (4.0)	15 685 (3.7)	317 (3.7)
New Jersey	27 495 (6.9)	1583 (9.9)	18 357 (7.5)	1112 (10.3)	24 597 (6.9)	1542 (9.0)	32 025 (7.6)	997 (11.7)
New Mexico	5676 (1.4)	108 (0.7)	3373 (1.4)	78 (0.7)	4040 (1.1)	116 (0.7)	6263 (1.5)	63 (0.7)
New York	59 456 (14.8)	4041 (25.2)	37 294 (15.2)	2667 (24.7)	56 240 (15.7)	3958 (23.1)	62 501 (14.9)	2240 (26.3)
Texas	62 310 (15.6)	1444 (9.0)	38 480 (15.6)	918 (8.5)	54 960 (15.4)	1464 (8.5)	54 931 (13.1)	571 (6.7)
Utah	6115 (1.5)	108 (0.7)	2997 (1.2)	65 (0.6)	2960 (0.8)	92 (0.5)	9032 (2.2)	65 (0.8)
Seattle	15 734 (3.9)	436 (2.7)	7853 (3.2)	292 (2.7)	13 026 (3.6)	583 (3.4)	17 679 (4.2)	216 (2.5)
Yost Index state quintile								
First (lowest SES)	53 502 (13.4)	6619 (41.2)	41 114 (16.7)	4485 (41.5)	58 512 (16.3)	7184 (41.9)	59 213 (14.1)	4203 (49.4)
Second	73 312 (18.3)	3558 (22.1)	51 239 (20.8)	2424 (22.4)	76 211 (21.3)	4000 (23.3)	76 409 (18.2)	1731 (20.4)
Third	84 807 (21.2)	2663 (16.6)	53 889 (21.9)	1798 (16.6)	79 904 (22.3)	2744 (16.0)	86 867 (20.7)	1161 (13.7)
Fourth	91 151 (22.8)	2037 (12.7)	52 026 (21.1)	1338 (12.4)	76 111 (21.3)	2056 (12.0)	92 847 (22.1)	878 (10.3)
Fifth (highest SES)	97 679 (24.4)	1187 (7.4)	47 822 (19.4)	762 (7.1)	67 260 (18.8)	1172 (6.8)	104 625 (24.9)	532 (6.3)

Abbreviations: ESKD, end-stage kidney disease; HUD, US Department of Housing and Urban Development; NA, not applicable; NSCLC, non–small cell lung cancer; SEER, Surveillance, Epidemiology, and End Results Program; SES, socioeconomic status.

^a^ Individuals had new diagnoses of breast cancer, prostate cancer, colorectal cancer, or NSCLC at ages 66 to 95 years, separately according to receipt and timing of federal housing assistance relative to cancer diagnosis. All individuals meeting inclusion criteria are included here, before propensity score matching was performed. Data source is the SEER-Medicare database and HUD.

^b^ Cohort of individuals was continuously enrolled in a HUD federal housing assistance program for at least 6 months before and up to the month of diagnosis.

^c^ Includes individuals with a marital status other than married including those who are single, divorced, separated, widowed, unmarried, or live with a domestic partner, and those whose marital status is unknown.

^d^ Large metropolitan includes counties in metropolitan areas of 250 000 to 1 million residents or more, small or medium metropolitan includes counties in metropolitan areas of fewer than 250 000 residents, and rural includes all other counties with smaller populations.

^e^ Percentages in this category may not add up to 100%, as those with missing Part D data were retained as a separate category not included here.

**Figure 1.**
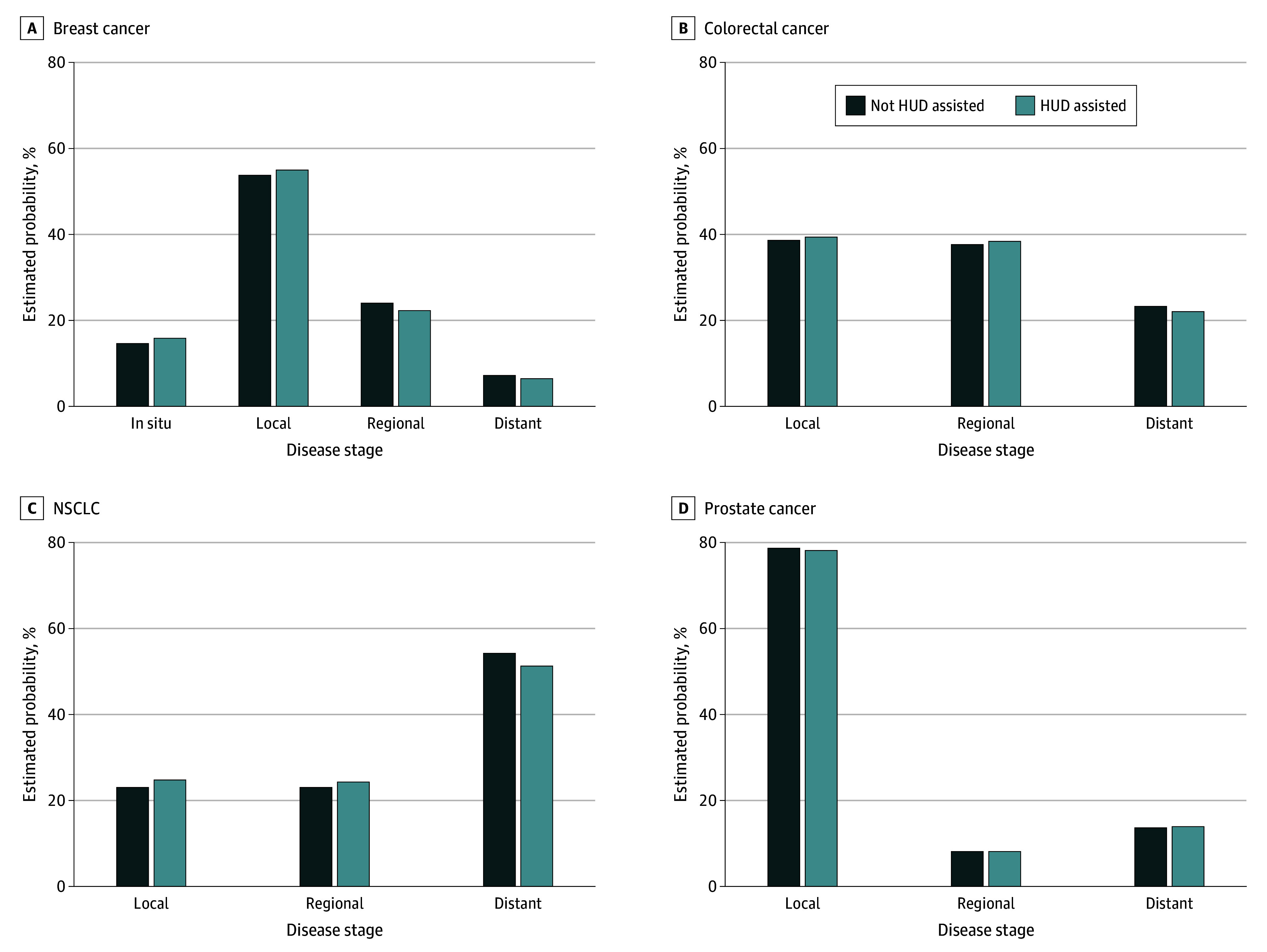
Estimated Probabilities of Stage at Diagnosis by US Department of Housing and Urban Development (HUD) Status Estimated probabilities of diagnosis of in situ (breast only), localized, regional, or distant cancer are shown according to whether individuals were receiving housing assistance for at least 6 months before and up to the month of diagnosis. Estimated probabilities were estimated from multinomial regression models, separately by cancer type, on the propensity score matched cohort, adjusting for all covariates included in the propensity score matching. NSCLC indicates non–small cell lung cancer.

In adjusted analyses, women with housing assistance were significantly less likely than those without housing assistance to receive a diagnosis of regional disease (3598 patients [22.4%] vs 11 650 patients [24.2%]; adjusted OR [aOR], 0.86; 95% CI,0.81-0.93; *P* < .001) or distant breast cancer (1071 patients [6.7%] vs 3485 patients [7.2%]; aOR, 0.85; 95% CI, 0.82-0.90; *P* < .001) vs localized disease. Individuals with housing assistance were significantly less likely than those without housing assistance to receive a diagnosis of distant vs localized colorectal cancer (2398 patients [22.2%] vs 7562 patients [23.3%]; aOR, 0.90; 95% CI, 0.83-0.98; *P* = .01) and distant vs localized NSCLC (8810 patients [51.4%] vs 27 901 patients [54.2%]; aOR, 0.83; 95% CI, 0.79-0.86; *P* < .001) (Table 2). There were no significant differences in stage of prostate cancer diagnosis among men with and without housing assistance in adjusted models. Estimated probabilities are presented in Figure 1.

**Table 2.** Association Between Federal Housing Assistance and Stage at Cancer Diagnosis

Cancer type and stage at diagnosis	Total No. of patients	Patients, No. (%)		aOR (95% CI)^b^	*P* value
		Not HUD assisted	HUD assisted^a^		
Breast cancer					
No. of patients		48 192	16 064		
Stage at diagnosis					
In situ	9643	7063 (14.7)	2580 (16.1)	1.04 (1.00-1.09)	.08
Localized	34 809	25 994 (53.9)	8815 (54.9)	1.00 [Reference]	
Regional	15 248	11 650 (24.2)	3598 (22.4)	0.86 (0.81-0.93)	<.001
Distant	4556	3485 (7.2)	1071 (6.7)	0.85 (0.82-0.90)	<.001
Colorectal cancer					
No. of patients		32 421	10 807		
Stage at diagnosis					
Localized	16 865	12 595 (38.8)	4270 (39.5)	1.00 [Reference]	
Regional	16 403	12 264 (37.8)	4139 (38.3)	0.99 (0.91-1.07)	.83
Distant	9960	7562 (23.3)	2398 (22.2)	0.90 (0.83-0.98)	.01
NSCLC					
No. of patients		51 468	17 156		
Stage at diagnosis					
Localized	15 993	11 800 (22.9)	4193 (24.4)	1.00 [Reference]	
Regional	15 920	11 767 (22.9)	4153 (24.2)	0.99 (0.97-1.02)	.55
Distant	36 711	27 901 (54.2)	8810 (51.4)	0.83 (0.79-0.86)	<.001
Prostate cancer					
No. of patients		25 515	8505		
Stage at diagnosis					
Localized	26 701	20 052 (78.6)	6649 (78.2)	1.00 [Reference]	
Regional	2734	2055 (8.1)	679 (8.0)	1.02 (0.90-1.17)	.74
Distant	4585	3408 (13.4)	1177 (13.8)	1.03 (0.92-1.16)	.62

Abbreviations: aOR, adjusted odds ratio; HUD, US Department of Housing and Urban Development; NSCLC, non–small cell lung cancer.

^a^ Individuals continuously enrolled in a HUD federal housing assistance program for at least 6 months before and up to the month of diagnosis were included in the analysis.

^b^ aORs for the association between having HUD assistance at diagnosis and cancer stage at diagnosis were estimated using multinomial regression. The avgcomparisons() function in the marginaleffects R package was used to estimate the average treatment effect on the treated for pairwise comparisons of interest. Models fully adjust for all covariates included in the matching.

### Secondary Analyses

**Figure 2.**
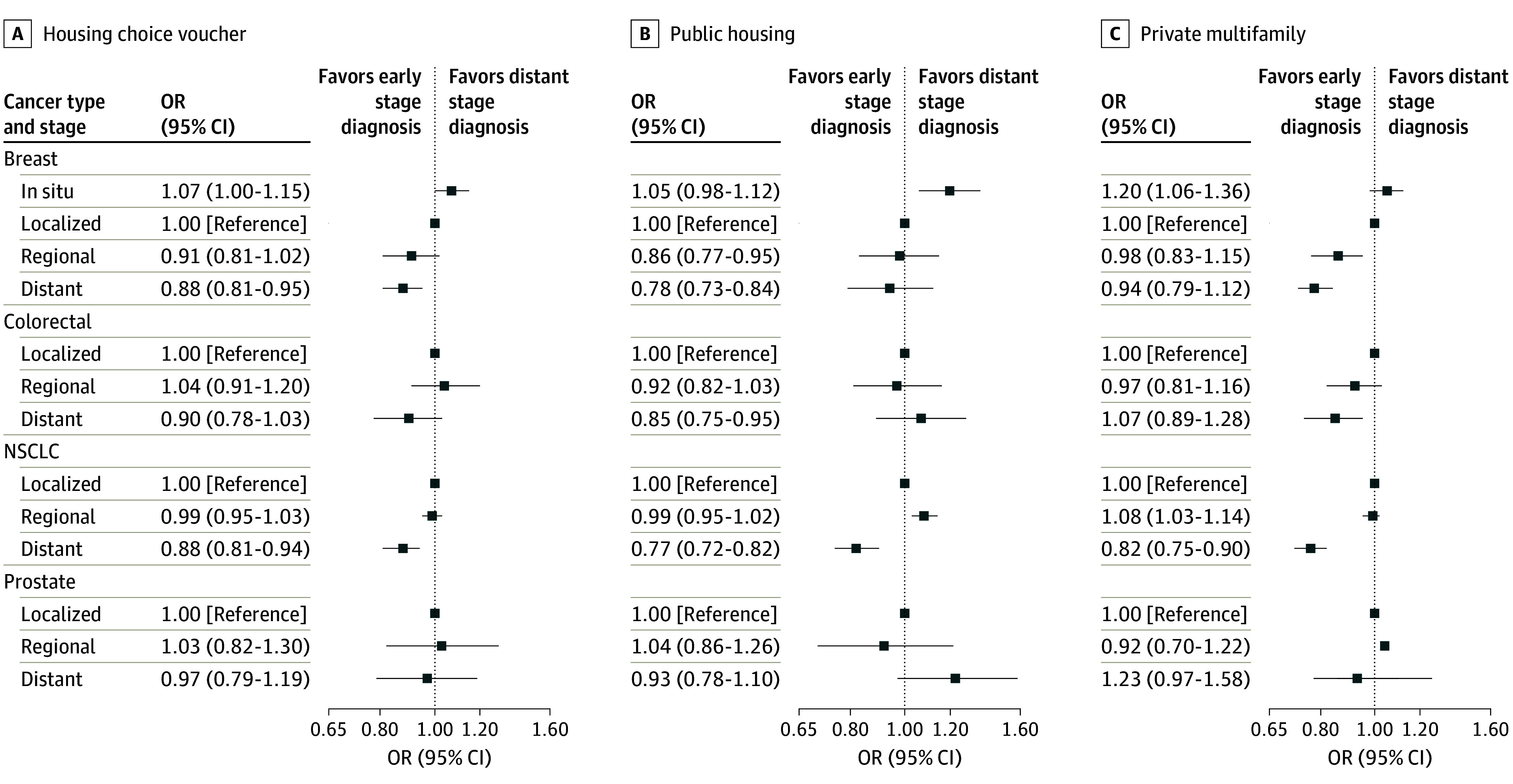
Association Between Stage at Diagnosis and Receipt of Federal Housing Assistance, Separately by Type of Assistance Forest plot of odds ratios (ORs) for the association between the Surveillance, Epidemiology, and End Results registry summary stage at diagnosis and whether individuals were receiving housing assistance through a Housing Choice voucher, private multifamily housing assistance, or public housing voucher at the time of diagnosis, separately by cancer type. ORs were estimated from multinomial regression models, separately by cancer type, using the propensity score matched cohort, adjusting for all covariates included in the propensity score matching. NSCLC indicates non–small cell lung cancer.

For each cancer type, the associations between housing assistance and stage at diagnosis did not vary by race or ethnicity, type of Medicare insurance (fee-for-service vs Medicare Advantage), sex (colorectal cancer and NSCLC), neighborhood SES, or year of diagnosis (eFigures 3-8 in Supplement 1). We observed differences in associations across the types of housing assistance (Figure 2; eTable 2 in Supplement 1). Across all 3 types of housing assistance, there were lower odds of distant stage NSCLC for assisted vs unassisted individuals. Individuals with Housing Choice vouchers and those living in multifamily housing also had significantly lower odds of distant stage breast cancer compared with unassisted individuals.

### Sensitivity Analyses

Limiting the sample to SEER registries that report American Joint Committee on Cancer data, we continued to observe significantly lower odds of later stage breast cancer and NSCLC and lower odds of late-stage prostate cancer among patients with housing assistance at diagnosis (eTable 3 in Supplement 1). Restricting the sample to fee-for-service Medicare and including comorbidity scores did not change the associations between housing assistance and stage (eTable 4 in Supplement 1).

We calculated E-values to determine the strength of our findings relative to unmeasured confounding (eTable 5 in Supplement 1). E-values ranged from 1.46 to 1.70, suggesting our results are moderately robust to unmeasured confounding.

### Estimated Cost Differences

Approximately 2.3 million older adults live in very-low-income renter households and pay more than one-half of their income to rent and/or live in severely inadequate conditions.^24^ Extending housing assistance to these individuals would be associated with approximately 378 fewer cases of regional or distant stage breast cancer, colorectal cancer, and NSCLC, with an estimated cost of cancer care savings of approximately $15 million in the first year after diagnosis (eTable 6 in Supplement 1).

## Discussion

To our knowledge, this cohort study provides the first national estimates of the association between federal housing assistance, a subsidy received by 1.8 million older adults, and stage at cancer diagnosis among Medicare beneficiaries. We found that housing assistance was associated with lower odds of distant vs localized stage disease for breast cancer (15% lower odds), colorectal cancer (10% lower odds), and NSCLC (17% lower odds). These differences were consistent across Medicare Advantage and fee-for-service beneficiaries, racial or ethnic groups, age groups, sex, and neighborhood SES.

Differences in the associations between housing assistance and stage types may reflect a range of factors, including likelihood of cancer screening, especially in the setting of changing clinical guidelines; however, prior research using survey data did not find differences in self-reported breast cancer screening among individuals with and without housing assistance.^25^ Other mechanisms, including early evaluation of symptoms, social stressors, and environmental exposures like cigarette smoke, may help explain the relationship between housing and stage across different cancer types. Further research is required to determine the potential pathways of observed associations.

The most consistent associations with earlier-stage diagnoses were observed for residents of multifamily and Housing Choice voucher recipients. While all types of housing assistance limit out-of-pocket spending on rent and utilities, potentially freeing up income for health-related expenses, the characteristics of the housing and services offered can vary across programs. Multifamily housing assistance programs frequently have on-site service coordinators, who screen residents for social and medical needs and make resources and programming available. Because units in multifamily and public housing are typically clustered in specific buildings, there may be additional benefits of reducing social isolation^26^ and modifying awareness and social norms around cancer screening and early evaluation of symptoms. Public housing may be colocated with associated health centers and other services that support access to cancer care. Housing vouchers, which enable recipients to rent homes on the private market, do not provide service coordination or colocated health services but do give residents access to a wider selection of neighborhoods, which might improve access to health care and facilitate earlier cancer detection.

Although all types of housing assistance require homes to meet certain quality standards, exposure to cancer-related risk factors may differ.^27^ For example, many housing authorities instituted smoke-free policies in public housing in advance of a 2018 federal mandate. Multifamily buildings and landlords that accept vouchers may also limit smoking in their housing units but are not required to do so. Different types of housing assistance may also provide differential access to cancer-related risk factors concerning diet and exercise (eg, availability of healthy foods and safe environments for exercise). It is possible that observed differences by type of housing assistance may reflect underlying differences in the populations served by the different programs. The current findings are consistent with prior research showing different health benefits across types of housing assistance.^28^

This study did not find significant variation in associations across racial or ethnic group or area-level SES. However, because housing assistance serves a disproportionate number of individuals from minoritized racial and ethnic groups and individuals living in high-poverty areas, housing assistance may help reduce disparities in cancer stage at diagnosis.

Cost savings from earlier stage at diagnosis alone were modest, especially considering the $55 billion overall cost of housing assistance in the US in 2023,^29^ suggesting that these short-term potential costs savings are alone unlikely to justify substantial investments in housing assistance. However, these savings likely underestimate the full economic impact, including longer-term costs, and make an important contribution to growing evidence on the ways that housing assistance may benefit health more broadly and reduce inequities.^30,31,32,33^

### Limitations

This study has limitations. First, the linkage between SEER-Medicare and HUD data may be incomplete. The extent to which people with HUD assistance are not identified, and thus included in the comparison group, would likely bias results toward the null. Second, HUD data do not consistently document when an individual leaves housing assistance, which may lead to misclassification error. Nonetheless, our approach of creating episodes of housing assistance is aligned with HUD guidance.^17^ Third, generalizability of findings is limited to SEER states and regions and to older adults. Fourth, there is potential for unobserved confounding. For example, we are unable to account for household income and other factors associated with eligibility or for an eligible individual’s ability to navigate the often difficult process of obtaining housing assistance; these factors may be associated with health-related behaviors. Fifth, the study does not directly examine factors such as cancer screening or timely evaluation of early cancer symptoms, which may contribute to observed differences and is an important direction for future research. We accounted for changing screening guidelines in secondary analyses, even though clinical practice was often slow to respond and there is uncertainty around incorporating race and ethnicity into prostate cancer screening recommendations.^34^ Sixth, housing assistance is administered by local public housing authorities, which are heterogeneous in their programmatic oversight, housing stock, and other features. Correspondingly, the association between housing assistance and cancer outcomes may also vary by locality. Seventh, individuals with missing stage at diagnosis or covariates were excluded; low levels of missingness suggest minimal impact on generalizability or internal validity (eTable 7 in Supplement 1). Eighth, cost estimates are meant to be exploratory.

## Conclusions

In this cohort study of older adults in the US with cancer, we found that receipt of housing assistance was associated with earlier-stage breast cancer, colorectal cancer, and NSCLC diagnoses. Given that only about 1 in 4 eligible individuals currently receive housing assistance, a potential benefit of extending housing assistance may be earlier-stage diagnoses of these cancers. With minoritized racial and ethnic groups experiencing disproportionate burdens of housing security, housing assistance may also help reduce long-standing cancer inequities. As the population ages and health needs grow, future studies must consider meaningful policy-focused interventions in addressing housing insecurity.

## Notes

Supplement 1. **eFigure 1.** CONSORT diagram illustrating the cohort selection process for the HOMES study **eFigure 2.** Effect of propensity score matching on covariate balance between individuals with and without HUD assistance **eFigure 3.** Association between SEER summary stage at diagnosis and receipt of HUD assistance for subgroups defined according to SEX **eFigure 4.** Association between SEER summary stage at diagnosis and receipt of HUD assistance for subgroups defined according to RACE AND ETHNICITY **eFigure 5.** Association between SEER summary stage at diagnosis and receipt of HUD assistance for subgroups defined according to INSURANCE STATUS **eFigure 6.** Association between SEER summary stage at diagnosis and receipt of HUD assistance for subgroups defined according to YOST INDEX **eFigure 7.** Association between SEER summary stage at diagnosis and receipt of HUD assistance for subgroups defined according to YEAR OF DIAGNOSIS **eFigure 8.** Association between SEER summary stage at diagnosis and receipt of HUD assistance for subgroups defined according to AGE GROUP **eTable 1.** Descriptive summary of individual characteristics measured at cancer diagnosis for the matched cohort **eTable 2.** Association between SEER summary stage at diagnosis and receipt of HUD assistance by type of housing assistance **eTable 3.** Association between AJCC stage at diagnosis and receipt of HUD assistance **eTable 4.** Association between SEER summary stage at diagnosis and Housing Choice voucher status, with and without comorbidity score **eTable 5.** E-values for the primary analysis of the association between receipt of federal housing assistance on stage at cancer diagnosis, for results that were found to be statistically significant **eTable 6.** Estimated total cost savings in the first year after diagnosis with HUD assistance compared to no assistance **eTable 7.** Missing stage at diagnosis and covariates by cancer type

Supplement 2.
Data Sharing Statement
