# Supplementary material for: Federal Housing Assistance and Stage at Cancer Diagnosis Among Older Adults in the US
Source: JAMA Netw Open. 2025 Oct 8;8(10):e2536281. doi: 10.1001/jamanetworkopen.2025.36281 (PMC12508992; doi:10.1001/jamanetworkopen.2025.36281)
Supplement: Supplement 1. — eFigure 1. CONSORT diagram illustrating the cohort selection process for the HOMES study eFigure 2. Effect of propensity score matching on covariate balance between individuals with and without HUD assistance eFigure 3. Association between SEER summary stage at diagnosis and receipt of HUD assistance for subgroups defined according to SEX eFigure 4. Association between SEER summary stage at diagnosis and receipt of HUD assistance for subgroups defined according to RACE AND ETHNICITY eFigure 5. Association between SEER summary stage at diagnosis and receipt of HUD assistance for subgroups defined according to INSURANCE STATUS eFigure 6. Association between SEER summary stage at diagnosis and receipt of HUD assistance for subgroups defined according to YOST INDEX eFigure 7. Association between SEER summary stage at diagnosis and receipt of HUD assistance for subgroups defined according to YEAR OF DIAGNOSIS eFigure 8. Association between SEER summary stage at diagnosis and receipt of HUD assistance for subgroups defined according to AGE GROUP eTable 1. Descriptive summary of individual characteristics measured at cancer diagnosis for the matched cohort eTable 2. Association between SEER summary stage at diagnosis and receipt of HUD assistance by type of housing assistance eTable 3. Association between AJCC stage at diagnosis and receipt of HUD assistance eTable 4. Association between SEER summary stage at diagnosis and Housing Choice voucher status, with and without comorbidity score eTable 5. E-values for the primary analysis of the association between receipt of federal housing assistance on stage at cancer diagnosis, for results that were found to be statistically significant eTable 6. Estimated total cost savings in the first year after diagnosis with HUD assistance compared to no assistance eTable 7. Missing stage at diagnosis and covariates by cancer type [file jamanetwopen-e2536281-s001.pdf]

Supplemental Online Content

Pollack CE, Blackford AL, Craig TK, et al. Federal housing assistance and earlier-stage cancer diagnosis among older adults in the US. *JAMA Netw Open.* 2025;8(10):e2536281. doi:10.1001/jamanetworkopen.2025.36281

eFigure 1. CONSORT diagram illustrates the cohort selection process for the HOMES study

eFigure 2. Effect of propensity score matching on covariate balance between individuals with and without HUD assistance

eFigure 3. Association between SEER summary stage at diagnosis and receipt of HUD assistance for subgroups defined according to SEX

eFigure 4. Association between SEER summary stage at diagnosis and receipt of HUD assistance for subgroups defined according to RACE AND ETHNICITY

eFigure 5. Association between SEER summary stage at diagnosis and receipt of HUD assistance for subgroups defined according to INSURANCE STATUS

eFigure 6. Association between SEER summary stage at diagnosis and receipt of HUD assistance for subgroups defined according to YOST INDEX

eFigure 7. Association between SEER summary stage at diagnosis and receipt of HUD assistance for subgroups defined according to YEAR OF DIAGNOSIS

eFigure 8. Association between SEER summary stage at diagnosis and receipt of HUD assistance for subgroups defined according to AGE GROUP

eTable1. Descriptive summary of individual characteristics measured at cancer diagnosis for the matched cohort

eTable 2. Association between SEER summary stage at diagnosis and receipt of HUD assistance by type of housing assistance

eTable 3. Association between AJCC stage at diagnosis and receipt of HUD assistance

eTable 4. Association between SEER summary stage at diagnosis and housing choice voucher status, with and without comorbidity score

eTable 5. E-values for the primary analysis of the association between receipt of federal housing assistance on stage at cancer diagnosis, for results that were found to be statistically significant

eTable 6. Estimated total cost savings in the first year after diagnosis with HUD assistance compared to no assistance

eTable 7. Missing stage at diagnosis and covariates by cancer type

This supplemental material has been provided by the authors to give readers additional information about their work.

© 2025 Pollack CE et al. *JAMA Network Open*.

© 2025 Pollack CE et al. *JAMA Network Open*.

eFigure 1. CONSORT diagram illustrates the cohort selection process for the HOMES study.

The diagram presents the flow of participants at each stage, detailing the numbers of participants excluded for not meeting the inclusion criteria. Arrows indicate transitions between each step, from the initial match of the SEER-Medicare linkage to HUD federal housing assistance data to the final cohort for analysis, with numbers of participants at each step shown in parentheses.

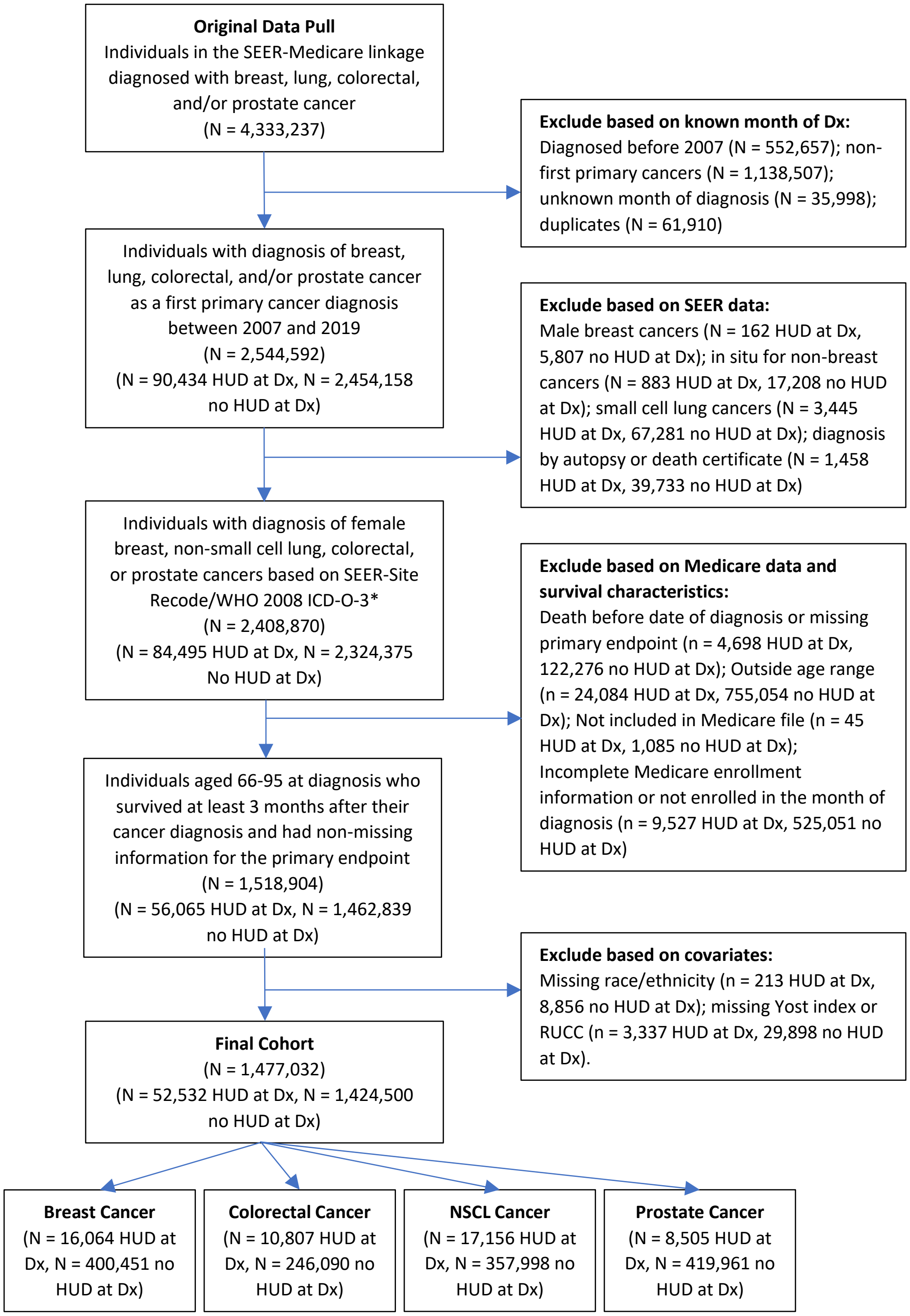

*SEER-Site Recode/WHO 2008 International Classification of Diseases for Oncology (ICD-O-3) codes for these diagnoses were: 26000 (breast), 22030 excluding histology codes 8041-8045 (non-small cell lung), 21041-21049/21051/21052/21060 (colorectal), and 28010 (prostate).

© 2025 Pollack CE et al. *JAMA Network Open*.

eFigure 2. Effect of propensity score matching on covariate balance between individuals with and without HUD assistance

Love Plots generated using R package 'cobalt' illustrate the balance of covariates after propensity score matching between individuals who were and were not receiving HUD assistance for at least 6 months before and up to the month of diagnosis. The plot visualizes the standardized mean differences (SMD) of covariates included after matching, with the y-axis representing individual covariates and the x-axis showing the standardized mean difference. The solid vertical line at zero represents perfect balance and the dashed lines at SMD = 0.1 and -0.1 represent the commonly used threshold indicating good balance in the covariate distribution between groups, with points closer to this line suggesting better covariate adjustment.

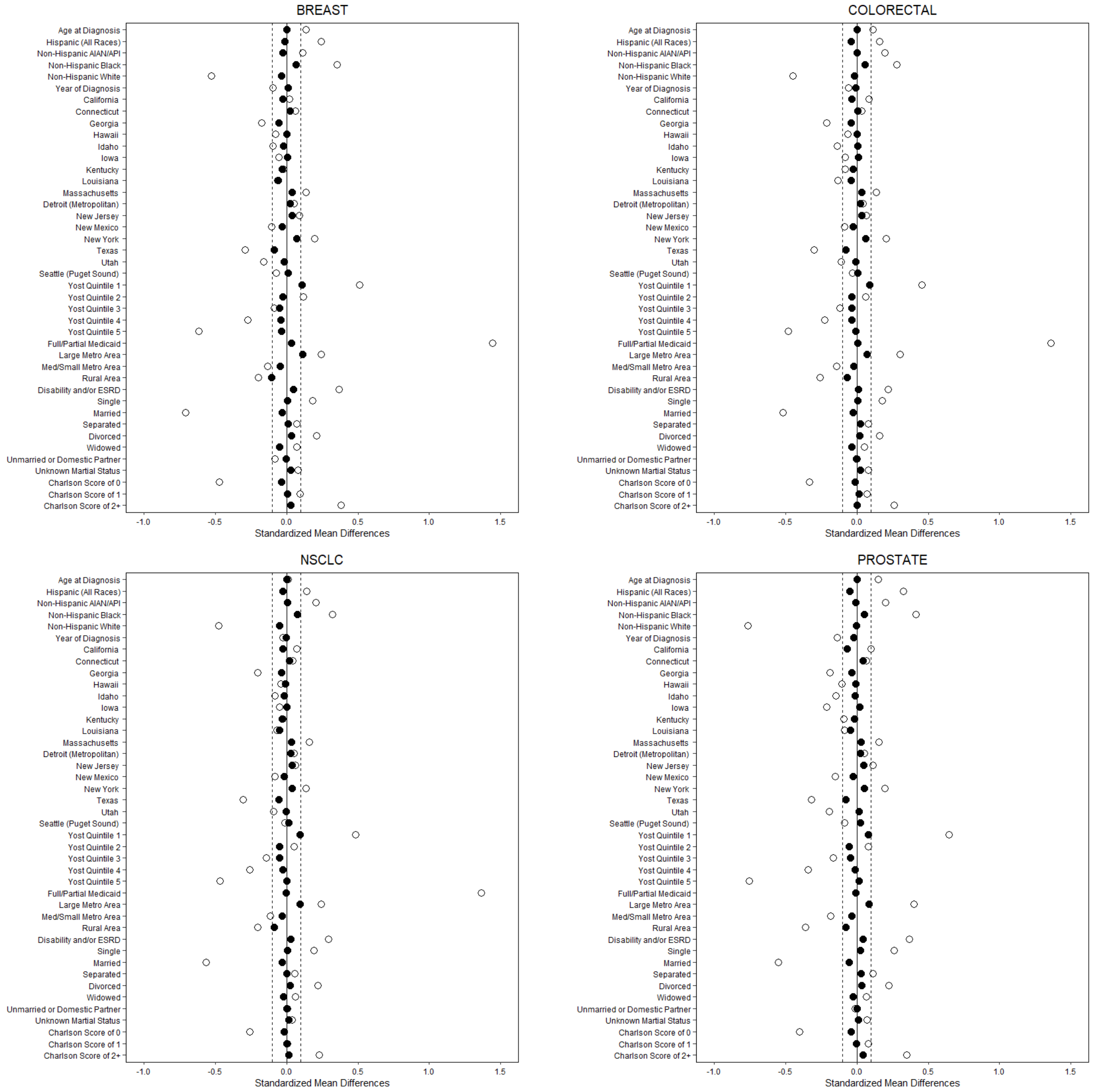

© 2025 Pollack CE et al. *JAMA Network Open*.

eFigure 3. Association between SEER summary stage at diagnosis and receipt of HUD assistance for subgroups defined according to SEX

Forest plot of odds ratios for the association between SEER summary stage at diagnosis and whether individuals were receiving HUD assistance for at least 6 months before and up to the month of diagnosis, separately by cancer type (colorectal and lung cancers only) for subgroups defined according to sex (males and females). Plot shows the subgroup-specific odds ratios and 95% confidence intervals, with variation in box size corresponding to subgroup sample size. P-values are for tests of heterogeneity between groups defined by sex (interaction analysis). Significance is adjusted using a Bonferroni threshold based on the total number of interactions tests by each cancer type: (breast ($p < 0.05/4 = 0.0125$), colorectal ($p < 0.05/5 = 0.01$), NSCL ($p < 0.05/6 = 0.008$), and prostate ($p < 0.05/5= 0.01$).

Cancer	Subgroup	Stage	Not HUD Assisted N (%)	HUD Assisted N (%)	Odds Ratio [95% CI]	Interaction P Value
Colorectal	Women	Localized	8324 (39.1)	2849 (40.2)	1.0 (ref)	0.3
		Regional	8105 (38.1)	2715 (38.3)	0.97 [0.88, 1.07]	
		Distant	4835 (22.7)	1524 (21.5)	0.88 [0.80, 0.97]	
	Men	Localized	4309 (38.6)	1421 (38.2)	1.0 (ref)	
		Regional	4224 (37.9)	1424 (38.3)	1.05 [0.91, 1.20]	
		Distant	2624 (23.5)	874 (23.5)	1.01 [0.88, 1.16]	
NSCLC	Women	Localized	7762 (24.7)	2777 (26.6)	1.0 (ref)	0.59
		Regional	7180 (22.9)	2516 (24.1)	0.97 [0.94, 1.00]	
		Distant	16426 (52.4)	5163 (49.4)	0.81 [0.76, 0.85]	
	Men	Localized	4020 (20.0)	1416 (21.1)	1.0 (ref)	
		Regional	4724 (23.5)	1637 (24.4)	0.97 [0.93, 1.01]	
		Distant	11356 (56.5)	3647 (54.4)	0.85 [0.79, 0.91]	

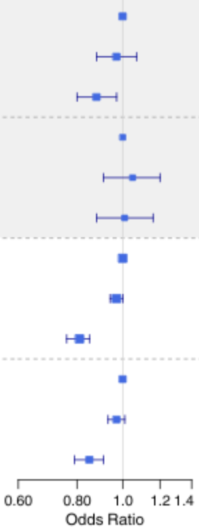

© 2025 Pollack CE et al. *JAMA Network Open*.

eFigure 4. Association between SEER summary stage at diagnosis and receipt of HUD assistance for subgroups defined according to RACE AND ETHNICITY

Forest plot of odds ratios for the association between SEER summary stage at diagnosis and whether individuals were receiving HUD assistance for at least 6 months before and up to the month of diagnosis, separately by cancer type for subgroups defined according to race and ethnicity (AAPI = Asian American or Pacific Islander) at diagnosis. Plot shows the subgroup-specific odds ratio and 95% confidence intervals, with variation in box size corresponding to subgroup sample size. P-values are for tests of heterogeneity between groups defined by race/ethnicity (interaction analysis). Significance is adjusted using a Bonferroni threshold based on the total number of interactions tests by each cancer type: (breast ($p < 0.05/4 = 0.0125$), colorectal ($p < 0.05/5 = 0.01$), NSCL ($p < 0.05/6 = 0.008$), and prostate ($p < 0.05/5 = 0.01$).

Cancer	Subgroup	Stage	Not HUD Assisted N (%)	HUD Assisted N (%)	Odds Ratio [95% CI]	Interaction P Value
Breast	Hispanic (All Races)	In situ	990 (16.3)	581 (19.2)	1.13 [0.98, 1.30]	0.03
		Localized	3185 (52.6)	1631 (53.8)	1.0 (ref)	
		Regional	1502 (24.8)	659 (21.7)	0.83 [0.70, 0.98]	
		Distant	381 (6.3)	159 (5.2)	0.80 [0.66, 0.96]	
	Non−Hispanic Black	In situ	1498 (18.2)	788 (19.1)	1.07 [0.96, 1.19]	
		Localized	3972 (48.2)	2003 (48.6)	1.0 (ref)	
		Regional	2060 (25.0)	995 (24.1)	0.95 [0.82, 1.09]	
		Distant	714 (8.7)	336 (8.2)	0.93 [0.80, 1.09]	
	Non−Hispanic AAPI	In situ	392 (18.9)	241 (23.2)	1.21 [0.93, 1.57]	
		Localized	1086 (52.4)	564 (54.3)	1.0 (ref)	
		Regional	472 (22.8)	201 (19.4)	0.73 [0.54, 0.97]	
		Distant	124 (6.0)	32 (3.1)	0.44 [0.33, 0.59]	
	Non−Hispanic White	In situ	2854 (12.2)	965 (12.3)	0.93 [0.88, 1.00]	
		Localized	13284 (56.6)	4587 (58.6)	1.0 (ref)	
		Regional	5484 (23.4)	1733 (22.1)	0.86 [0.78, 0.95]	
		Distant	1853 (7.9)	540 (6.9)	0.79 [0.74, 0.85]	
		Localized	1338 (38.6)	703 (40.5)	1.0 (ref)	
Colorectal	Hispanic (All Races)	Regional	1374 (39.7)	637 (36.7)	0.84 [0.68, 1.03]	0.27
		Distant	753 (21.7)	394 (22.7)	0.98 [0.79, 1.22]	
		Localized	1790 (37.3)	920 (38.3)	1.0 (ref)	
	Non−Hispanic Black	Regional	1675 (34.9)	853 (35.6)	0.98 [0.82, 1.18]	
		Distant	1333 (27.8)	626 (26.1)	0.86 [0.72, 1.03]	
		Localized	867 (38.8)	428 (38.2)	1.0 (ref)	
	Non−Hispanic AAPI	Regional	909 (40.7)	479 (42.8)	1.05 [0.81, 1.37]	
		Distant	459 (20.5)	212 (18.9)	0.90 [0.68, 1.17]	
		Localized	6514 (39.3)	2207 (40.0)	1.0 (ref)	
	Non−Hispanic White	Regional	6298 (38.0)	2160 (39.1)	1.01 [0.90, 1.13]	
		Distant	3754 (22.7)	1155 (20.9)	0.87 [0.77, 0.97]	

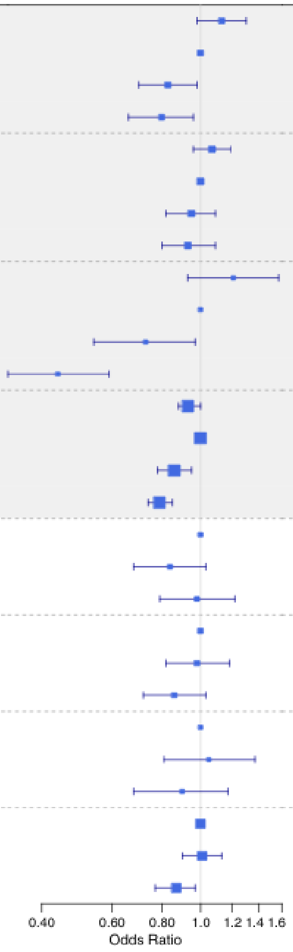

© 2025 Pollack CE et al. *JAMA Network Open*.

Cancer	Subgroup	Stage	Not HUD Assisted N (%)	HUD Assisted N (%)	Odds Ratio [95% CI]	Interaction P Value
NSCLC	Hispanic (All Races)	Localized	866 (21.8)	486 (24.5)	1.0 (ref)	0.89
		Regional	902 (22.7)	484 (24.4)	0.94 [0.84, 1.04]	
		Distant	2199 (55.4)	1014 (51.1)	0.75 [0.65, 0.87]	
	Non−Hispanic Black	Localized	1606 (19.6)	874 (21.3)	1.0 (ref)	
		Regional	1937 (23.6)	1043 (25.4)	1.00 [0.95, 1.06]	
		Distant	4664 (56.8)	2188 (53.3)	0.79 [0.72, 0.87]	
	Non−Hispanic AAPI	Localized	646 (19.0)	375 (22.0)	1.0 (ref)	
		Regional	668 (19.7)	349 (20.5)	0.86 [0.80, 0.93]	
		Distant	2085 (61.3)	980 (57.5)	0.71 [0.61, 0.83]	
	Non−Hispanic White	Localized	6698 (24.0)	2438 (26.2)	1.0 (ref)	
		Regional	6640 (23.8)	2267 (24.4)	0.92 [0.89, 0.95]	
		Distant	14577 (52.2)	4600 (49.4)	0.80 [0.75, 0.84]	
Prostate	Hispanic (All Races)	Localized	3181 (81.4)	1574 (80.5)	1.0 (ref)	0.69
		Regional	321 (8.2)	150 (7.7)	0.99 [0.73, 1.34]	
		Distant	405 (10.4)	231 (11.8)	1.26 [0.96, 1.66]	
	Non−Hispanic Black	Localized	4518 (78.8)	2268 (79.1)	1.0 (ref)	
		Regional	415 (7.2)	205 (7.1)	0.98 [0.77, 1.26]	
		Distant	802 (14.0)	395 (13.8)	0.95 [0.76, 1.18]	
	Non−Hispanic AAPI	Localized	1111 (77.3)	563 (78.0)	1.0 (ref)	
		Regional	153 (10.6)	74 (10.2)	0.86 [0.55, 1.34]	
		Distant	174 (12.1)	85 (11.8)	0.86 [0.56, 1.31]	
	Non−Hispanic White	Localized	6697 (76.0)	2229 (75.9)	1.0 (ref)	
		Regional	759 (8.6)	250 (8.5)	0.99 [0.80, 1.22]	
		Distant	1358 (15.4)	459 (15.6)	0.98 [0.81, 1.19]	

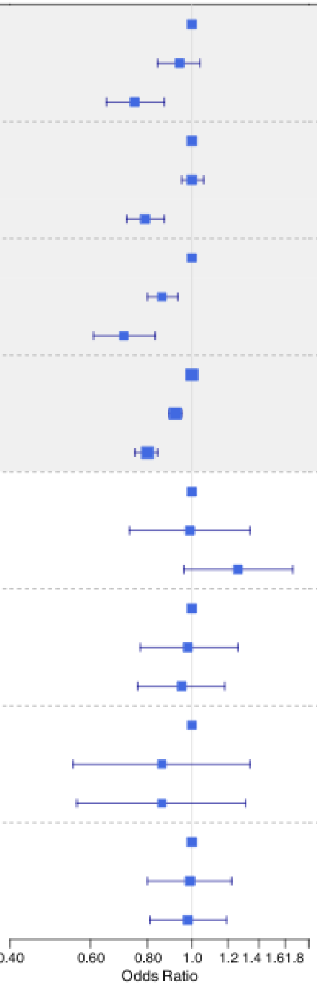

© 2025 Pollack CE et al. *JAMA Network Open*.

eFigure 5. Association between SEER summary stage at diagnosis and receipt of HUD assistance for subgroups defined according to INSURANCE STATUS

Forest plot of odds ratios for the association between SEER summary stage at diagnosis and whether individuals were receiving HUD assistance for at least 6 months before and up to the month of diagnosis, separately by cancer type for subgroups defined according to Medicaid program enrolled in the month of diagnosis (fee-for-service Medicare or a Medicare Advantage program). Plot shows the subgroup-specific odds ratios and 95% confidence intervals, with variation in box size corresponding to subgroup sample size. P-values are for tests of heterogeneity between groups defined by type of Medicare coverage (interaction analysis). Significance is adjusted using a Bonferroni threshold based on the total number of interactions tests by each cancer type: (breast ($p < 0.05/4 = 0.0125$), colorectal ($p < 0.05/5 = 0.01$), NSCL ($p < 0.05/6 = 0.008$), and prostate ($p < 0.05/5= 0.01$).

Cancer	Subgroup	Stage	Not HUD Assisted N (%)	HUD Assisted N (%)	Odds Ratio [95% CI]	Interaction P Value
Breast	Fee-For-Service	In situ	4210 (13.7)	1568 (15.3)	1.06 [0.99, 1.13]	>0.99
		Localized	16403 (53.2)	5627 (54.8)	1.0 (ref)	
		Regional	7682 (24.9)	2337 (22.8)	0.83 [0.76, 0.90]	
		Distant	2515 (8.2)	738 (7.2)	0.79 [0.72, 0.87]	
	Medicare Advantage	In situ	2921 (16.8)	1012 (17.5)	1.02 [0.95, 1.10]	
		Localized	9461 (54.4)	3188 (55.0)	1.0 (ref)	
		Regional	3925 (22.6)	1261 (21.8)	0.92 [0.82, 1.04]	
		Distant	1075 (6.2)	333 (5.7)	0.87 [0.81, 0.94]	
Colorectal	Fee-For-Service	Localized	8620 (38.1)	2977 (39.5)	1.0 (ref)	0.5
		Regional	8640 (38.2)	2884 (38.3)	0.94 [0.86, 1.04]	
		Distant	5354 (23.7)	1677 (22.2)	0.85 [0.77, 0.94]	
	Medicare Advantage	Localized	3873 (39.5)	1293 (39.6)	1.0 (ref)	
		Regional	3732 (38.1)	1255 (38.4)	1.01 [0.88, 1.17]	
		Distant	2202 (22.5)	721 (22.1)	0.97 [0.83, 1.12]	
NSCLC	Fee-For-Service	Localized	7660 (22.2)	2830 (24.6)	1.0 (ref)	0.38
		Regional	7947 (23.0)	2752 (23.9)	0.92 [0.89, 0.94]	
		Distant	18962 (54.9)	5941 (51.6)	0.76 [0.72, 0.80]	
	Medicare Advantage	Localized	3865 (22.9)	1363 (24.2)	1.0 (ref)	
		Regional	3923 (23.2)	1401 (24.9)	1.01 [0.97, 1.06]	
		Distant	9111 (53.9)	2869 (50.9)	0.82 [0.76, 0.88]	
Prostate	Fee-For-Service	Localized	12450 (77.0)	4181 (77.6)	1.0 (ref)	>0.99
		Regional	1378 (8.5)	439 (8.1)	0.94 [0.80, 1.10]	
		Distant	2333 (14.4)	767 (14.2)	0.94 [0.81, 1.08]	
	Medicare Advantage	Localized	7545 (80.7)	2468 (79.2)	1.0 (ref)	
		Regional	738 (7.9)	240 (7.7)	1.08 [0.86, 1.34]	
		Distant	1071 (11.4)	410 (13.1)	1.18 [0.97, 1.44]	

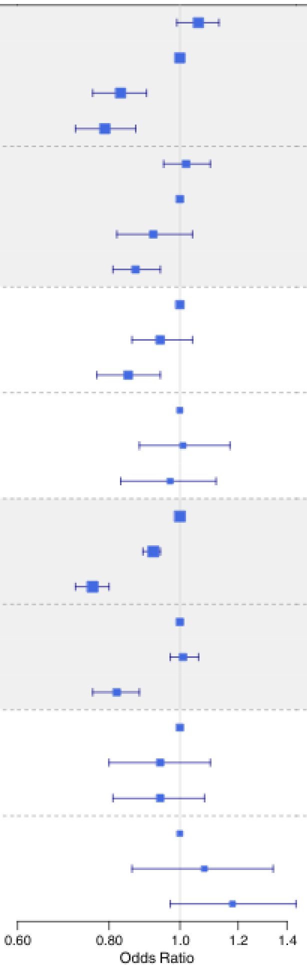

© 2025 Pollack CE et al. *JAMA Network Open*.

eFigure 6. Association between SEER summary stage at diagnosis and receipt of HUD assistance for subgroups defined according to YOST INDEX

Forest plot of odds ratios for the association between SEER summary stage at diagnosis and whether individuals were receiving HUD assistance for at least 6 months before and up to the month of diagnosis, separately by cancer type for subgroups defined according to quintiles of their area-level state-based Yost index (values 1 to 5, with higher values representing areas of higher socioeconomic status) at diagnosis. Quintiles 4 and 5 were combined prior to analysis due to small sample sizes in these groups. Plot shows the subgroup-specific odds ratio and 95% confidence intervals, with variation in box size corresponding to subgroup sample size. P-values are for tests of heterogeneity between groups defined by Yost Index quintiles (interaction analysis). Significance is adjusted using a Bonferroni threshold based on the total number of interactions tests by each cancer type: (breast ($p < 0.05/4 = 0.0125$), colorectal ($p < 0.05/5 = 0.01$), NSCL ($p < 0.05/6 = 0.008$), and prostate ($p < 0.05/5 = 0.01$).

Cancer	Subgroup	Stage	Not HUD Assisted N (%)	HUD Assisted N (%)	Odds Ratio [95% CI]	Interaction P Value
Breast	Yost Quintile 1	In situ	2999 (15.1)	1184 (17.9)	1.19 [1.10, 1.29]	0.01
		Localized	10489 (52.8)	3498 (52.8)	1.0 (ref)	
		Regional	4884 (24.6)	1515 (22.9)	0.88 [0.79, 0.99]	
		Distant	1485 (7.5)	422 (6.4)	0.80 [0.72, 0.89]	
	Yost Quintile 3	In situ	1114 (13.9)	384 (14.4)	1.00 [0.90, 1.12]	
		Localized	4374 (54.8)	1487 (55.8)	1.0 (ref)	
		Regional	1934 (24.2)	600 (22.5)	0.86 [0.73, 1.02]	
		Distant	567 (7.1)	192 (7.2)	0.96 [0.85, 1.07]	
	Yost Quintile 2	In situ	1543 (14.5)	496 (13.9)	0.91 [0.83, 1.00]	
		Localized	5857 (54.9)	1994 (56.0)	1.0 (ref)	
		Regional	2496 (23.4)	823 (23.1)	0.92 [0.79, 1.06]	
		Distant	778 (7.3)	245 (6.9)	0.86 [0.78, 0.95]	
	Yost Quintiles 4–5	In situ	1499 (15.5)	516 (16.0)	0.97 [0.88, 1.08]	
		Localized	5349 (55.3)	1836 (56.9)	1.0 (ref)	
		Regional	2187 (22.6)	660 (20.5)	0.83 [0.71, 0.96]	
		Distant	637 (6.6)	212 (6.6)	0.92 [0.83, 1.03]	
		Localized	5134 (38.2)	1747 (39.0)	1.0 (ref)	
Colorectal	Yost Quintile 1	Regional	5070 (37.7)	1647 (36.7)	0.94 [0.83, 1.06]	0.32
		Distant	3251 (24.2)	1091 (24.3)	0.95 [0.84, 1.07]	
		Localized	2123 (39.4)	695 (38.7)	1.0 (ref)	
	Yost Quintile 3	Regional	2041 (37.8)	710 (39.5)	1.09 [0.90, 1.33]	
		Distant	1230 (22.8)	393 (21.9)	0.96 [0.79, 1.17]	
		Localized	2806 (38.6)	975 (40.2)	1.0 (ref)	
	Yost Quintile 2	Regional	2843 (39.1)	979 (40.4)	0.98 [0.83, 1.16]	
		Distant	1623 (22.3)	470 (19.4)	0.77 [0.65, 0.91]	
		Localized	2505 (39.8)	853 (40.6)	1.0 (ref)	
	Yost Quintiles 4–5	Regional	2411 (38.3)	803 (38.2)	0.95 [0.79, 1.14]	
		Distant	1384 (22.0)	444 (21.1)	0.90 [0.75, 1.08]	

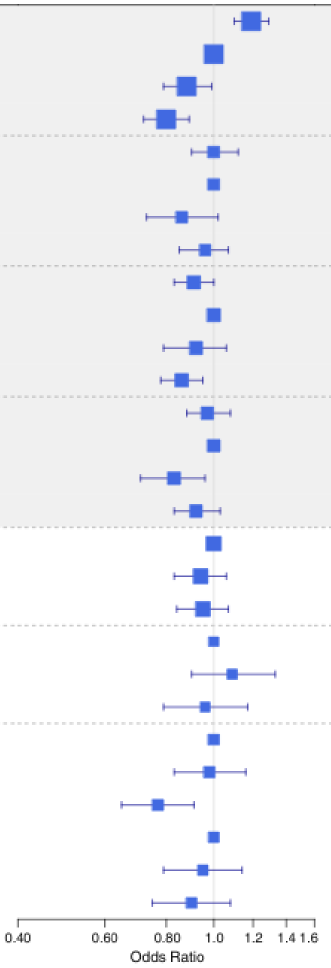

© 2025 Pollack CE et al. *JAMA Network Open*.

Cancer	Subgroup	Stage	Not HUD Assisted N (%)	HUD Assisted N (%)	Odds Ratio [95% CI]	Interaction P Value
NSCLC	Yost Quintile 1	Localized	4586 (21.3)	1739 (24.2)	1.0 (ref)	0.64
		Regional	4906 (22.8)	1753 (24.4)	0.93 [0.90, 0.97]	
		Distant	12057 (56.0)	3692 (51.4)	0.71 [0.67, 0.76]	
	Yost Quintile 3	Localized	1876 (22.8)	657 (23.9)	1.0 (ref)	
		Regional	1933 (23.5)	689 (25.1)	1.02 [0.96, 1.09]	
		Distant	4423 (53.7)	1398 (50.9)	0.85 [0.77, 0.94]	
	Yost Quintile 2	Localized	2717 (22.6)	977 (24.4)	1.0 (ref)	
		Regional	2792 (23.3)	970 (24.2)	0.95 [0.90, 0.99]	
		Distant	6491 (54.1)	2053 (51.3)	0.80 [0.74, 0.88]	
	Yost Quintiles 4–5	Localized	2292 (23.7)	820 (25.4)	1.0 (ref)	
		Regional	2175 (22.5)	741 (23.0)	0.93 [0.88, 0.98]	
		Distant	5217 (53.9)	1667 (51.6)	0.84 [0.76, 0.92]	
Prostate	Yost Quintile 1	Localized	10005 (79.4)	3342 (79.5)	1.0 (ref)	>0.99
		Regional	993 (7.9)	312 (7.4)	0.95 [0.78, 1.15]	
		Distant	1607 (12.7)	549 (13.1)	0.98 [0.82, 1.16]	
	Yost Quintile 3	Localized	2698 (77.5)	876 (75.5)	1.0 (ref)	
		Regional	284 (8.2)	103 (8.9)	1.24 [0.88, 1.74]	
		Distant	501 (14.4)	182 (15.7)	1.13 [0.84, 1.53]	
	Yost Quintile 2	Localized	4049 (78.0)	1363 (78.7)	1.0 (ref)	
		Regional	450 (8.7)	124 (7.2)	0.79 [0.59, 1.06]	
		Distant	694 (13.4)	244 (14.1)	0.98 [0.76, 1.28]	
	Yost Quintiles 4–5	Localized	3250 (76.8)	1068 (75.7)	1.0 (ref)	
		Regional	419 (9.9)	140 (9.9)	1.07 [0.79, 1.44]	
		Distant	561 (13.3)	202 (14.3)	1.07 [0.81, 1.40]	

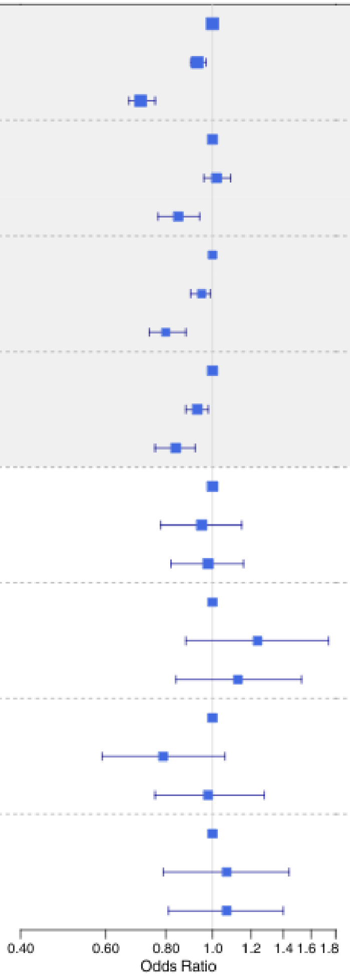

© 2025 Pollack CE et al. *JAMA Network Open*.

eFigure 7. Association between SEER summary stage at diagnosis and receipt of HUD assistance for subgroups defined according to YEAR OF DIAGNOSIS

Forest plot of odds ratios for the association between SEER summary stage at diagnosis and whether individuals were receiving HUD assistance for at least 6 months before and up to the month of diagnosis, separately by cancer type for subgroups defined according to year of diagnosis (prostate and NSCLC only). Plot shows the subgroup-specific odds ratios and 95% confidence intervals, with variation in box size corresponding to subgroup sample size. P-values are for tests of heterogeneity between groups defined by year of diagnosis (interaction analysis). Significance is adjusted using a Bonferroni threshold based on the total number of interactions tests by each cancer type: (breast ($p < 0.05/4 = 0.0125$), colorectal ($p < 0.05/5 = 0.01$), NSCL ($p < 0.05/6 = 0.008$), and prostate ($p < 0.05/5 = 0.01$).

Cancer	Subgroup	Stage	Not HUD Assisted N (%)	HUD Assisted N (%)	Odds Ratio [95% CI]	Interaction P Value
Prostate	2007 to 2011	Localized	9276 (83.9)	3110 (84.4)	1.0 (ref)	>0.99
		Regional	739 (6.7)	240 (6.5)	0.94 [0.75, 1.18]	
		Distant	1046 (9.5)	337 (9.1)	0.87 [0.70, 1.07]	
	2012 to 2017	Localized	8106 (74.6)	2696 (74.4)	1.0 (ref)	
		Regional	1043 (9.6)	320 (8.8)	0.92 [0.77, 1.12]	
		Distant	1717 (15.8)	606 (16.7)	1.00 [0.85, 1.19]	
	2018 to 2019	Localized	2527 (70.4)	843 (70.5)	1.0 (ref)	
		Regional	405 (11.3)	119 (9.9)	0.90 [0.66, 1.23]	
		Distant	656 (18.3)	234 (19.6)	1.05 [0.79, 1.38]	
NSCLC	2007 to 2014	Localized	6430 (20.5)	2292 (21.9)	1.0 (ref)	0.69
		Regional	7384 (23.6)	2598 (24.9)	0.98 [0.95, 1.02]	
		Distant	17539 (55.9)	5561 (53.2)	0.82 [0.78, 0.87]	
	2015 to 2019	Localized	5226 (26.0)	1901 (28.4)	1.0 (ref)	
		Regional	4513 (22.4)	1555 (23.2)	0.94 [0.90, 0.97]	
		Distant	10376 (51.6)	3249 (48.5)	0.79 [0.74, 0.85]	

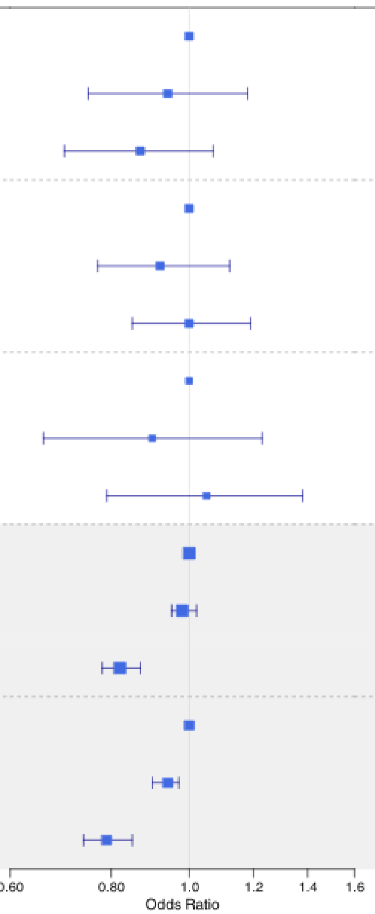

© 2025 Pollack CE et al. *JAMA Network Open*.

eFigure 8. Association between SEER summary stage at diagnosis and receipt of HUD assistance for subgroups defined according to AGE GROUP

Forest plot of odds ratios for the association between SEER summary stage at diagnosis and whether individuals were receiving HUD assistance for at least 6 months before and up to the month of diagnosis, separately by cancer type for subgroups defined according to age (66 to 74, 75 to 95 years) at diagnosis. Plot shows the subgroup-specific odds ratio and 95% confidence intervals, with variation in box size corresponding to subgroup sample size. P-values are for tests of heterogeneity between groups defined by age (interaction analysis). Significance is adjusted using a Bonferroni threshold based on the total number of interactions tests by each cancer type: (breast ($p < 0.05/4 = 0.0125$), colorectal ($p < 0.05/5 = 0.01$), NSCL ($p < 0.05/6 = 0.008$), and prostate ($p < 0.05/5 = 0.01$).

Cancer	Subgroup	Stage	Not HUD Assisted N (%)	HUD Assisted N (%)	Odds Ratio [95% CI]	Interaction P Value
Breast	66 to <75	In situ	4163 (17.5)	1502 (18.9)	1.01 [0.94, 1.08]	>0.99
		Localized	12516 (52.5)	4298 (54.1)	1.0 (ref)	
		Regional	5621 (23.6)	1674 (21.1)	0.81 [0.73, 0.89]	
		Distant	1532 (6.4)	470 (5.9)	0.84 [0.78, 0.92]	
	75 to 95	In situ	2944 (12.1)	1078 (13.3)	1.06 [0.98, 1.14]	
		Localized	13354 (54.8)	4517 (55.6)	1.0 (ref)	
		Regional	6042 (24.8)	1924 (23.7)	0.90 [0.82, 0.99]	
		Distant	2020 (8.3)	601 (7.4)	0.83 [0.74, 0.92]	
Colorectal	66 to <75	Localized	4880 (39.4)	1621 (39.3)	1.0 (ref)	0.5
		Regional	4547 (36.7)	1534 (37.2)	1.02 [0.90, 1.17]	
		Distant	2951 (23.8)	971 (23.5)	0.96 [0.84, 1.09]	
	75 to 95	Localized	7778 (38.8)	2649 (39.6)	1.0 (ref)	
		Regional	7705 (38.4)	2605 (39.0)	0.98 [0.89, 1.09]	
		Distant	4560 (22.8)	1427 (21.4)	0.87 [0.79, 0.97]	
NSCLC	66 to <75	Localized	5416 (22.7)	1984 (24.9)	1.0 (ref)	0.87
		Regional	5758 (24.1)	2022 (25.4)	0.94 [0.91, 0.97]	
		Distant	12724 (53.2)	3960 (49.7)	0.76 [0.72, 0.81]	
	75 to 95	Localized	6109 (22.2)	2209 (24.0)	1.0 (ref)	
		Regional	6041 (21.9)	2131 (23.2)	0.96 [0.93, 0.99]	
		Distant	15420 (55.9)	4850 (52.8)	0.79 [0.74, 0.83]	
Prostate	66 to <75	Localized	12153 (80.9)	4100 (81.9)	1.0 (ref)	0.05
		Regional	1487 (9.9)	447 (8.9)	0.86 [0.72, 1.02]	
		Distant	1381 (9.2)	460 (9.2)	0.94 [0.79, 1.11]	
	75 to 95	Localized	7778 (74.1)	2549 (72.9)	1.0 (ref)	
		Regional	633 (6.0)	232 (6.6)	1.16 [0.94, 1.41]	
		Distant	2083 (19.8)	717 (20.5)	1.04 [0.88, 1.22]	

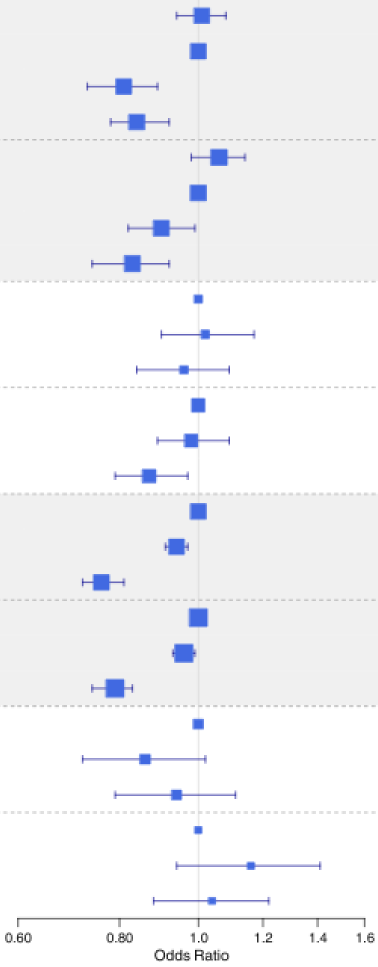

© 2025 Pollack CE et al. *JAMA Network Open*.

eTable 1. Descriptive summary of individual characteristics measured at cancer diagnosis for the matched cohort

Characteristics of individuals in the HOMES study diagnosed with breast, prostate, colorectal, or non-small cell lung cancer at age 66 to 95, separately according to receipt and timing of HUD assistance relative to cancer diagnosis for the matched cohort. Individuals who were not HUD assisted represent those included in the final analysis, after propensity score matching in a 3:1 ratio using a nearest-neighbor distance specification. All variables shown in the table were included in the matching, with an exact specification for age.

	Breast Cancer		Colorectal Cancer		Non-Small Cell Lung Cancer		Prostate Cancer	
	HUD Assisted[a]	Not HUD Assisted	HUD Assisted[a]	Not HUD Assisted	HUD Assisted[a]	Not HUD Assisted	HUD Assisted[a]	Not HUD Assisted
	N = 16064	N = 48192	N = 10807	N = 32421	N = 17156	N = 51468	N = 8505	N = 25515
Age, y - mean (SD)	76.0 (6.8)		78.0 (7.2)		76.4 (6.7)		74.5 (6.1)	
Sex – %								
Male	0 (0.0)	0 (0.0)	3719 (34.4)	11535 (35.6)	6700 (39.1)	21169 (41.1)	8505 (100.0)	25515 (100.0)
Female	16064 (100.0)	48192 (100.0)	7088 (65.6)	20886 (64.4)	10456 (60.9)	30299 (58.9)	0 (0.0)	0 (0.0)
Race and Ethnicity – n (%)								
Hispanic (All races)	3030 (18.9)	9227 (19.1)	1734 (16.0)	5460 (16.8)	1984 (11.6)	6186 (12.0)	1955 (23.0)	6379 (25.0)
NH AIAN[b]	48 (0.3)	207 (0.4)	33 (0.3)	172 (0.5)	57 (0.3)	238 (0.5)	21 (0.2)	107 (0.4)
NH AAPI[c]	1039 (6.5)	3656 (7.6)	1119 (10.4)	3514 (10.8)	1705 (9.9)	5435 (10.6)	723 (8.5)	2381 (9.3)
NH Black	4122 (25.7)	10789 (22.4)	2399 (22.2)	6442 (19.9)	4105 (23.9)	10512 (20.4)	2868 (33.7)	8154 (32.0)
NH White	7825 (48.7)	24313 (50.5)	5522 (51.1)	16833 (51.9)	9305 (54.2)	29097 (56.5)	2938 (34.5)	8494 (33.3)
Married[d] – n (%)	1296 (8.1)	4121 (8.6)	1348 (12.5)	4283 (13.2)	1953 (11.4)	6298 (12.2)	1591 (18.7)	5102 (20.0)
Metropolitan Residence[e] – n (%)								
Large Metro	14007 (87.2)	40634 (84.3)	9319 (86.2)	27196 (83.9)	14482 (84.4)	42005 (81.6)	7541 (88.7)	22238 (87.2)
Med/Small Metro	923 (5.7)	3136 (6.5)	610 (5.6)	2008 (6.2)	1146 (6.7)	3752 (7.3)	463 (5.4)	1486 (5.8)
Rural	1134 (7.1)	4422 (9.2)	878 (8.1)	3217 (9.9)	1528 (8.9)	5711 (11.1)	501 (5.9)	1791 (7.0)
Original Reason for Medicare Entitlement – n (%)								
Age	12680 (78.9)	39277 (81.5)	9030 (83.6)	27345 (84.3)	13018 (75.9)	40006 (77.7)	6604 (77.6)	20331 (79.7)
Disability ± ESRD[f]	3384 (21.1)	8915 (18.5)	1777 (16.4)	5076 (15.6)	4138 (24.2)	11462 (22.2)	1901 (22.3)	5184 (20.3)
Medicaid Enrolled – %	11593 (72.2)	34378 (71.3)	7899 (73.1)	23827 (73.5)	12540 (73.1)	37701 (73.3)	6151 (72.3)	18465 (72.4)
Part D Low Income Cost Sharing[g] – n (%)								
Fully Subsidized	11695 (73.0)	33767 (70.3)	7965 (73.9)	23211 (71.7)	12673 (74.0)	36877 (71.8)	6206 (73.4)	18039 (71.0)

© 2025 Pollack CE et al. *JAMA Network Open*.

Eligible, Not Receiving	869 (5.4)	619 (1.3)	550 (5.1)	421 (1.3)	870 (5.1)	693 (1.3)	439 (5.2)	293 (1.2)
Not Eligible	3451 (21.5)	13677 (28.5)	2270 (21.0)	8719 (27.0)	3592 (21.0)	13809 (26.9)	1813 (21.4)	7060 (27.8)
Enrolled in MA[h] – n (%)	5794 (36.1)	17179 (35.6)	3269 (30.2)	9772 (30.1)	5633 (32.8)	16514 (32.1)	3118 (36.7)	9213 (36.1)
SEER Registry – n (%)								
California	3241 (20.2)	10595 (22.0)	2434 (22.5)	7780 (24.0)	3356 (19.6)	10789 (21.0)	1721 (20.2)	5730 (22.5)
Connecticut	668 (4.2)	1807 (3.7)	386 (3.6)	1128 (3.5)	730 (4.3)	2023 (3.9)	360 (4.2)	896 (3.5)
Georgia	800 (5.0)	2835 (5.9)	508 (4.7)	1744 (5.4)	878 (5.1)	2943 (5.7)	505 (5.9)	1675 (6.6)
Hawaii	130 (0.8)	396 (0.8)	94 (0.9)	289 (0.9)	164 (1.0)	525 (1.0)	71 (0.8)	210 (0.8)
Idaho	88 (0.5)	308 (0.6)	38 (0.4)	143 (0.4)	93 (0.5)	301 (0.6)	32 (0.4)	95 (0.4)
Iowa	343 (2.1)	1010 (2.1)	246 (2.3)	771 (2.4)	398 (2.3)	1196 (2.3)	93 (1.1)	239 (0.9)
Kentucky	535 (3.3)	1766 (3.7)	363 (3.4)	1195 (3.7)	820 (4.8)	2691 (5.2)	187 (2.2)	596 (2.3)
Louisiana	449 (2.8)	1641 (3.4)	258 (2.4)	973 (3.0)	517 (3.0)	1882 (3.7)	294 (3.5)	990 (3.9)
Massachusetts	1558 (9.7)	4081 (8.5)	973 (9.0)	2576 (7.9)	1763 (10.3)	4752 (9.2)	773 (9.1)	2012 (7.9)
Detroit	532 (3.3)	1448 (3.0)	375 (3.5)	1023 (3.2)	682 (4.0)	1842 (3.6)	317 (3.7)	903 (3.5)
New Jersey	1583 (9.9)	4133 (8.6)	1112 (10.3)	2904 (9.0)	1542 (9.0)	4024 (7.8)	997 (11.7)	2674 (10.5)
New Mexico	108 (0.7)	410 (0.9)	78 (0.7)	296 (0.9)	116 (0.7)	413 (0.8)	63 (0.7)	249 (1.0)
New York	4041 (25.2)	10699 (22.2)	2667 (24.7)	7281 (22.5)	3958 (23.1)	11004 (21.4)	2240 (26.3)	6382 (25.0)
Texas	1444 (9.0)	5440 (11.3)	918 (8.5)	3267 (10.1)	1464 (8.5)	5116 (9.9)	571 (6.7)	2139 (8.4)
Utah	108 (0.7)	348 (0.7)	65 (0.6)	195 (0.6)	92 (0.5)	267 (0.5)	65 (0.8)	170 (0.7)
Seattle	436 (2.7)	1275 (2.6)	292 (2.7)	856 (2.6)	583 (3.4)	1700 (3.3)	216 (2.5)	555 (2.2)
YOST Index State Quintile – n (%)								
1 (lowest SES[i])	6619 (41.2)	17006 (35.3)	4485 (41.5)	12087 (37.3)	7184 (41.9)	19261 (37.4)	4203 (49.4)	11808 (46.3)
2	3558 (22.1)	11527 (23.9)	2424 (22.4)	7916 (24.4)	4000 (23.3)	13005 (25.3)	1731 (20.4)	5860 (23.0)
3	2663 (16.6)	9090 (18.9)	1798 (16.6)	5809 (17.9)	2744 (16.0)	9001 (17.5)	1161 (13.7)	3766 (14.8)
4	2037 (12.7)	6750 (14.0)	1338 (12.4)	4249 (13.1)	2056 (12.0)	6631 (12.9)	878 (10.3)	2661 (10.4)
5 (highest SES)	1187 (7.4)	3819 (7.9)	762 (7.1)	2360 (7.3)	1172 (6.8)	3570 (6.9)	532 (6.3)	1420 (5.6)

NOTE: Values are percentages except where noted

[a] Cohort of individuals continuously enrolled in a HUD federal housing assistance program for at least 6 months before and up to the month of diagnosis.

[b] American Indian and Alaska Native

© 2025 Pollack CE et al. *JAMA Network Open*.

[c] Asian American and Pacific Islander
[d] Individuals with a marital status other than "Married" include those who are single, divorced, separated, widowed, unmarried or live with a domestic partner, and those whose marital status is unknown.
[e] Large Metro includes counties in metro areas of 250,000 to 1 million or more; Small/Med Metro includes counties in metro areas of fewer than 250,000; Rural includes all other counties with smaller populations
[f] End-Stage Renal Disease
[g] Percentages in this category may not add up to 100%, as those with missing Part D data were retained as a separate category not included here.
[h] Medicare Advantage
[i] Census tract socioeconomic status

© 2025 Pollack CE et al. *JAMA Network Open*.

eTable 2. Association between SEER summary stage at diagnosis and receipt of HUD assistance by type of housing assistance

Frequency distribution of SEER summary stage at diagnosis and odds ratios for the association between stage and whether individuals were receiving HUD assistance for at least 6 months before and up to the month of diagnosis through a housing choice voucher (top section), private multifamily voucher (middle section), or public housing voucher (bottom section), separately by cancer type.

	HOUSING CHOICE VOUCHER			
	Not HUD Assisted	HUD Assisted[a]	Odds Ratio[b] [95% CI]	P
**BREAST CANCER**	(N = 17115)	(N = 5705)		
Stage at Diagnosis – %				
In Situ	2539 (14.8)	914 (16.0)	1.07 [1.00, 1.15]	0.07
Localized	9092 (53.1)	3066 (53.7)	1.0 (ref)	1.0 (ref)
Regional	4181 (24.4)	1324 (23.2)	0.91 [0.81, 1.02]	0.11
Distant	1303 (7.6)	401 (7.0)	0.88 [0.81, 0.95]	< 0.001
**COLORECTAL CANCER**	(N = 10704)	(N = 3568)		
Stage at Diagnosis – %				
Localized	4061 (37.9)	1362 (38.2)	1.0 (ref)	1.0 (ref)
Regional	4107 (38.4)	1414 (39.6)	1.04 [0.91, 1.20]	0.57
Distant	2536 (23.7)	792 (22.2)	0.90 [0.78, 1.03]	0.13
**NSCL CANCER**	(N = 17436)	(N = 5812)		
Stage at Diagnosis – %				
Localized	4126 (23.7)	1429 (24.6)	1.0 (ref)	1.0 (ref)
Regional	3925 (22.5)	1362 (23.4)	0.99 [0.95, 1.03]	0.72
Distant	9385 (53.8)	3021 (52.0)	0.88 [0.81, 0.94]	< 0.001
**PROSTATE CANCER**	(N = 7842)	(N = 2614)		
Stage at Diagnosis – %				
Localized	6031 (76.9)	2004 (76.7)	1.0 (ref)	1.0 (ref)
Regional	666 (8.5)	229 (8.8)	1.03 [0.82, 1.30]	0.77
Distant	1145 (14.6)	381 (14.6)	0.97 [0.79, 1.19]	0.77
	**PRIVATE MULTIFAMILY**			
	Not HUD Assisted	HUD Assisted[a]	Odds Ratio[b] [95% CI]	P
**BREAST CANCER**	(N = 22098)	(N = 7366)		
Stage at Diagnosis – %				
In Situ	3137 (14.2)	1144 (15.5)	1.05 [0.98, 1.12]	0.19
Localized	12197 (55.2)	4173 (56.7)	1.0 (ref)	1.0 (ref)
Regional	5125 (23.2)	1587 (21.5)	0.86 [0.77, 0.95]	0.003
Distant	1639 (7.4)	462 (6.3)	0.78 [0.73, 0.84]	< 0.001
**COLORECTAL CANCER**	(N = 15396)	(N = 5132)		
Stage at Diagnosis – %				
Localized	5997 (39.0)	2087 (40.7)	1.0 (ref)	1.0 (ref)
Regional	5906 (38.4)	1955 (38.1)	0.92 [0.82, 1.03]	0.15
Distant	3493 (22.7)	1090 (21.2)	0.85 [0.75, 0.95]	0.006
**NSCL CANCER**	(N = 23517)	(N = 7839)		
Stage at Diagnosis – %				

© 2025 Pollack CE et al. *JAMA Network Open*.

Localized	5340 (22.7)	1935 (24.7)	1.0 (ref)	1.0 (ref)
Regional	5277 (22.4)	1898 (24.2)	0.99 [0.95, 1.02]	0.43
Distant	12900 (54.9)	4006 (51.1)	0.77 [0.72, 0.82]	< 0.001
**PROSTATE CANCER**	(N = 11490)	(N = 3830)		
Stage at Diagnosis – %				
Localized	8974 (78.1)	3003 (78.4)	1.0 (ref)	1.0 (ref)
Regional	909 (7.9)	314 (8.2)	1.04 [0.86, 1.26]	0.69
Distant	1607 (14.0)	513 (13.4)	0.93 [0.78, 1.10]	0.38

PUBLIC HOUSING				
	Not HUD Assisted	HUD Assisted[a]	Odds Ratio[b] [95% CI]	P
**BREAST CANCER**	(N = 9135)	(N = 3045)		
Stage at Diagnosis – %				
In Situ	1373 (15.0)	530 (17.4)	1.20 [1.06, 1.36]	0.004
Localized	4897 (53.6)	1605 (52.7)	1.0 (ref)	1.0 (ref)
Regional	2186 (23.9)	701 (23.0)	0.98 [0.83, 1.15]	0.78
Distant	679 (7.4)	209 (6.9)	0.94 [0.79, 1.12]	0.48
**COLORECTAL CANCER**	(N = 6408)	(N = 2136)		
Stage at Diagnosis – %				
Localized	2510 (39.2)	832 (39.0)	1.0 (ref)	1.0 (ref)
Regional	2402 (37.5)	781 (36.6)	0.97 [0.81, 1.16]	0.73
Distant	1496 (23.3)	523 (24.5)	1.07 [0.89, 1.28]	0.50
**NSCL CANCER**	(N = 10656)	(N = 3552)		
Stage at Diagnosis – %				
Localized	2407 (22.6)	835 (23.5)	1.0 (ref)	1.0 (ref)
Regional	2448 (23.0)	908 (25.6)	1.08 [1.03, 1.14]	0.003
Distant	5801 (54.4)	1809 (50.9)	0.82 [0.75, 0.90]	<0.001
**PROSTATE CANCER**	(N = 6309)	(N = 2103)		
Stage at Diagnosis – %				
Localized	5076 (80.5)	1678 (79.8)	1.0 (ref)	1.0 (ref)
Regional	480 (7.6)	140 (6.7)	0.92 [0.70, 1.22]	0.56
Distant	753 (11.9)	285 (13.6)	1.23 [0.97, 1.58]	0.09

[a] Cohort of individuals continuously enrolled in a HUD federal housing assistance program for at least 6 months before and up to the month of diagnosis.

[b] Odds Ratios for the association between having HUD assistance at diagnosis and cancer stage at diagnosis estimated using multinomial regression. The avgcomparisons() function in the marginaleffects R package was used to estimate the ATT for pairwise comparisons of interest. Models fully adjust for all covariates included in the matching.

© 2025 Pollack CE et al. *JAMA Network Open*.

eTable 3. Association between AJCC stage at diagnosis and receipt of HUD assistance

Frequency distribution of American Joint Committee on Cancer (AJCC) stage at diagnosis and odds ratios for the association between stage and whether individuals were receiving HUD assistance for at least 6 months before and up to the month of diagnosis, separately by cancer type. Individuals were staged according to the AJCC edition in place at the time of diagnosis (6th edition: 2007 to 2009, 7th edition: 2010 to 2015, Derived SEER combined stage group: 2016 to 2017, Derived EOD stage group: 2018 to 2019).

	Not HUD Assisted	HUD Assisted[a]	Odds Ratio[b] [95% CI]	P
**BREAST CANCER**	(N = 25773)	(N = 8591)		
**Stage at Diagnosis - %**				
Stage 0	3889 (15.1)	1369 (15.9)	0.98 [0.92, 1.05]	0.64
Stage I	10378 (40.3)	3554 (41.4)	1.0 (ref)	
Stage II	6853 (26.6)	2255 (26.2)	0.92 [0.84, 1.01]	0.07
Stage III	2685 (10.4)	818 (9.5)	0.85 [0.77, 0.93]	<0.001
Stage IV	1968 (7.6)	595 (6.9)	0.82 [0.76, 0.88]	< 0.001
**COLORECTAL CANCER**	(N = 18153)	(N = 6051)		
**Stage at Diagnosis - %**				
Stage 0	514 (2.8)	156 (2.6)	0.86 [0.81, 0.93]	< 0.001
Stage I	3963 (21.8)	1329 (22.0)	1.0 (ref)	
Stage II	5071 (27.9)	1726 (28.5)	1.02 [0.91, 1.14]	0.72
Stage III	4609 (25.4)	1530 (25.3)	0.98 [0.88, 1.10]	0.73
Stage IV	3996 (22.0)	1310 (21.6)	0.95 [0.85, 1.07]	0.39
**NSCL CANCER**	(N = 28422)	(N = 9474)		
**Stage at Diagnosis - %**				
Stage I	6121 (21.5)	2223 (23.5)	1.0 (ref)	
Stage II	2157 (7.6)	738 (7.8)	0.97 [0.93, 1.00]	0.06
Stage III	6028 (21.2)	2109 (22.3)	0.99 [0.92, 1.05]	0.70
Stage IV	14116 (49.7)	4404 (46.5)	0.81 [0.75, 0.87]	< 0.001
**PROSTATE CANCER**	(N = 13821)	(N = 4607)		
**Stage at Diagnosis - %**				
Stage I	1944 (14.1)	675 (14.7)	1.0 (ref)	
Stage II	8377 (60.6)	2806 (60.9)	0.95 [0.86, 1.05]	0.27
Stage III	1168 (8.5)	367 (8.0)	0.87 [0.81, 0.94]	< 0.001
Stage IV	2332 (16.9)	759 (16.5)	0.87 [0.80, 0.95]	0.001

[a] Cohort of individuals continuously enrolled in a HUD federal housing assistance program for at least 6 months before and up to the month of diagnosis.

[b] Odds Ratios for the association between having HUD assistance at diagnosis and cancer stage at diagnosis estimated using multinomial regression. The avgcomparisons() function in the marginaleffects R package was used to estimate the ATT for pairwise comparisons of interest. Models fully adjust for all covariates included in the matching.

© 2025 Pollack CE et al. *JAMA Network Open*.

eTable 4. Association between SEER summary stage at diagnosis and housing choice voucher status, with and without comorbidity score

Frequency distribution of SEER summary stage at diagnosis and odds ratios for the association between stage and whether individuals were receiving HUD assistance for at least 6 months before and up to the month of diagnosis through a housing choice voucher with and without comorbidity score.

	Matched Cohort, With Comorbidity Score				*Matched Cohort, Without Comorbidity Score*			
	Not HUD Assisted	HUD Assisted [a]	OR[b] [95% CI]	P	Not HUD Assisted	HUD Assisted	OR [95% CI]	P
**BREAST CANCER**	(N=26925)	(N=8975)			(N=26925)	(N=8975)		
**Stage at Diagnosis - %**								
In Situ	3557 (13.2)	1352 (15.1)	1.09 [1.03, 1.16]	0.004	3612 (13.4)	1352 (15.1)	1.05 [0.99, 1.12]	0.09
Localized	14594 (54.2)	4968 (55.4)	1.0 (ref)		14500 (53.9)	4968 (55.4)	1.0 (ref)	
Regional	6653 (24.7)	2031 (22.6)	0.84 [0.77, 0.92]	< 0.001	6686 (24.8)	2031 (22.6)	0.82 [0.75, 0.90]	< 0.001
Distant	2121 (7.9)	624 (7.0)	0.81 [0.76, 0.86]	< 0.001	2127 (7.9)	624 (7.0)	0.79 [0.75, 0.84]	< 0.001
**COLORECTAL CANCER**	(N=19635)	(N=6545)			(N=19635)	(N=6545)		
**Stage at Diagnosis - %**								
Localized	7560 (38.5)	2609 (39.9)	1.0 (ref)		7584 (38.6)	2609 (39.9)	1.0 (ref)	
Regional	7492 (38.2)	2516 (38.4)	0.95 [0.86, 1.06]	0.38	7531 (38.4)	2516 (38.4)	0.95 [0.86, 1.05]	0.31
Distant	4583 (23.3)	1420 (21.7)	0.85 [0.76, 0.94]	0.002	4520 (23.0)	1420 (21.7)	0.86 [0.78, 0.96]	0.005
**NSCL CANCER**	(N=30138)	(N=10046)			(N=30138)	(N=10046)		
**Stage at Diagnosis - %**								
Localized	6904 (22.9)	2509 (25.0)	1.0 (ref)		6853 (22.7)	2509 (25.0)	1.0 (ref)	
Regional	6918 (23.0)	2403 (23.9)	0.95 [0.92, 0.98]	< 0.001	6890 (22.9)	2403 (23.9)	0.94 [0.91, 0.97]	< 0.001
Distant	16316 (54.1)	5134 (51.1)	0.79 [0.75, 0.84]	< 0.001	16395 (54.4)	5134 (51.1)	0.77 [0.73, 0.82]	< 0.001
**PROSTATE CANCER**	(N=13353)	(N=4451)			(N=13353)	(N=4451)		
**Stage at Diagnosis - %**								
Localized	10361 (77.6)	3445 (77.4)	1.0 (ref)		10396 (77.9)	3445 (77.4)	1.0 (ref)	
Regional	1102 (8.3)	354 (8.0)	1.00 [0.84, 1.20]	0.99	1090 (8.2)	354 (8.0)	1.02 [0.85, 1.22]	0.86
Distant	1890 (14.2)	652 (14.6)	1.04 [0.89, 1.22]	0.61	1867 (14.0)	652 (14.6)	1.06 [0.90, 1.24]	0.49

[a] Cohort of individuals continuously enrolled in a HUD federal housing assistance program for at least 6 months before and up to the month of diagnosis.

[b] Odds Ratios (OR) for the association between having HUD assistance at diagnosis and cancer stage at diagnosis estimated using multinomial regression. The avgcomparisons() function in the marginaleffects R package was used to estimate the ATT for pairwise comparisons of interest. Models fully adjust for all covariates included in the matching.

© 2025 Pollack CE et al. *JAMA Network Open*.

eTable 5. E-values for the primary analysis of the association between receipt of federal housing assistance on stage at cancer diagnosis, for results that were found to be statistically significant

Table of E-values for the primary analysis of the association between receipt of federal housing assistance on stage at cancer diagnosis, for results that were found to be statistically significant ($p < 0.05$). E-values provide an estimate of the required strength of an association between an unmeasured confounder and both the exposure (housing assistance) and the outcome (stage at cancer diagnosis) to explain away the observed association.

	Odds Ratio [95% CI]	E-Value [95% CI]
**BREAST CANCER**		
In Situ	1.04 [1.00, 1.09]	-
Localized	1.0 (ref)	
Regional	0.86 [0.81, 0.93]	1.60 [1.36, 1.77]
Distant	0.85 [0.82, 0.90]	1.63 [1.46, 1.74]
**COLORECTAL CANCER**		
Localized	1.0 (ref)	
Regional	0.99 [0.91, 1.07]	-
Distant	0.90 [0.83, 0.98]	1.46 [1.16, 1.70]
**NSCL CANCER**		
Localized	1.0 (ref)	
Regional	0.99 [0.97, 1.02]	-
Distant	0.83 [0.79, 0.86]	1.70 [1.60, 1.85]
**PROSTATE CANCER**		
Localized	1.0 (ref)	
Regional	1.02 [0.90, 1.17]	-
Distant	1.03 [0.92, 1.16]	-

E-values are calculated using the formula from *The use of the E-value for sensitivity analysis.* Chung, William T. et al. *Journal of Clinical Epidemiology*, Volume 163, 92 - 94.

E-value = OR + sqrt(OR x (1 - OR)) if OR > 1
E-value = (1/OR) + sqrt((1/OR) x ((1/OR) - 1) if OR < 1

© 2025 Pollack CE et al. *JAMA Network Open*.

eTable 6. Estimated total cost savings in the first year after diagnosis with HUD assistance compared to no assistance

Step-by-step calculations of estimated total cost savings in the first year following a cancer diagnosis that could be realized for providing older adults meeting HUD criteria for "worst-case housing needs" with HUD federal housing assistance continuously for at least 6 months before diagnosis compared to a similar cohort of individuals without housing assistance.

	**Breast Cancer**		**Colorectal Cancer**	**Non-Small Cell Lung Cancer**
Expected Number of New Cases Per Year[a]	6,073		3,567	6,428
Predicted Probability of Cancer Diagnosis[b]<br><br>With Housing Assistance<br>Without Housing Assistance	Distant v. Localized:<br><br>6.6%<br>7.3%	Regional v. Localized:<br><br>22.3%<br>24.2%	Distant v. Localized:<br><br>22.1%<br>23.3%	Distant v. Localized:<br><br>51.4%<br>54.2%
Average Risk Reduction	0.7%	1.9%	1.2%	2.8%
Number of Cases Prevented[c]	40	115	43	180
**Total Cases Prevented**	**378**			
Cost of Care Difference[d]	$37,600	$20,800	$80,100	$43,200
Cost Savings in First Year Following Diagnosis[e]	$ 1,504,000	$ 2,392,000	$ 3,444,300	$ 7,776,000
**Total Cost Savings in First Year Following Diagnosis**	**$15,116,300**			

[a] Number of new cases per year among estimated 2.3 million older adults with worst case housing needs and require housing assistance based on published incidence rates for female breast (455.3 per 100K), colorectal (155.1 per 100K), and non-small cell lung cancers (279.5 per 100K) in 2021; for female breast, 58% of population was assumed to be female based on U.S. Census Bureau published estimates for 2020. Sources: Worst Case Housing Needs 2023 Report to Congress, U.S. Department of Housing and Urban Development, https://www.huduser.gov/portal//portal/sites/default/files/pdf/Worst-Case-Housing-Needs-2023.pdf. SEER*Explorer: An interactive website for SEER cancer statistics [Internet]. Surveillance Research Program, National Cancer Institute; 2024 Apr 17. [updated: 2024 Jun 27; cited 2024 Oct 30]. Available from: https://seer.cancer.gov/statistics-network/explorer/. Data source(s): SEER Incidence Data, November 2023 Submission (1975-2021), SEER 22 registries

[b] Predicted probabilities of distant versus localized and regional versus localized cancers from multinomial regression models where receipt of housing assistance at the time of diagnosis was the main independent variable, adjusting for other factors

[c] Number of cases prevented = average risk reduction x expected number of new cases per year

[d] Difference in the per-patient cost of initial care (medical services + oral drugs) for a distant versus localized cancer diagnosis or a regional versus localized cancer diagnosis. Costs calculated from 2007 to 2013 Medicare claims for patients diagnosed with cancer between 2000 and 2012 and 65 years or older. Initial care included the first 12 months after diagnosis and reported using 2019 dollars. Based on: Mariotto AB, Enewold L, Zhao J, Zeruto CA, Yabroff KR. Medical Care Costs Associated with Cancer Survivorship in the United States. Cancer Epidemiol Biomarkers Prev. 2020 Jul;29(7):1304-1312.

[e] Cost savings = cost of care difference x number of cases prevented

© 2025 Pollack CE et al. *JAMA Network Open*.

eTable7. Missing stage at diagnosis and covariates by cancer type

Missing stage at diagnosis and covariates by cancer type

	Breast	Colorectal	NSCLC	Prostate
Missing Stage at Diagnosis	14,015 / 725,938 (1.9%)	23,472 / 401,845 (5.8%)	28,562 / 512,346 (5.6%)	58,540 / 767,615 (7.6%)
Missing Covariates: RUCC, Race Ethnicity or Yost Index	10,754 / 427,269 (2.5%)	6,161 / 263,058 (2.3%)	8,284 / 383,438 (2.3%)	16,673 / 445,139 (3.7%)

© 2025 Pollack CE et al. *JAMA Network Open*.
