# Supplementary material for: Federal Housing Assistance and Stage at Cancer Diagnosis Among Older Adults in the US
Source: JAMA Netw Open. 2025 Oct 8;8(10):e2536281. doi: 10.1001/jamanetworkopen.2025.36281 (PMC12508992; doi:10.1001/jamanetworkopen.2025.36281)
Supplement: Supplement 2. — Data Sharing Statement [file jamanetwopen-e2536281-s002.pdf]

Data Sharing Statement

Pollack. Federal Housing Assistance and Earlier-Stage Cancer Diagnosis Among Older Adults in the US. *JAMA Netw Open*. Published October 08, 2025.
doi:10.1001/jamanetworkopen.2025.36281

Data
Data available: No

Additional Information
Explanation for why data not available: Data are not publicly available. Information about how researchers can apply to use these data is provided on the National Cancer Institute's SEER-Medicare website
https://healthcaredelivery.cancer.gov/seermedicare/aboutdata/housing.html
